# Postsynthetic Modification of the Nonanuclear Node in a Zirconium Metal–Organic Framework for Photocatalytic Oxidation of Hydrocarbons

**DOI:** 10.1021/jacs.3c07237

**Published:** 2023-10-25

**Authors:** Rebecca Shu Hui Khoo, Christian Fiankor, Sizhuo Yang, Wenhui Hu, Chongqing Yang, Jingzhi Lu, Martha D. Morton, Xu Zhang, Yi Liu, Jier Huang, Jian Zhang

**Affiliations:** † The Molecular Foundry, Lawrence Berkeley National Laboratory, Berkeley, California 94720, United States; ‡ Department of Chemistry, University of Nebraska−Lincoln, Lincoln, Nebraska 68588, United States; § Department of Chemistry, Marquette University, Milwaukee, Wisconsin 53201, United States; ∥ Jiangsu Engineering Laboratory for Environment Functional Materials, Jiangsu Collaborative Innovation Center of Regional Modern Agriculture & Environmental Protection, School of Chemistry and Chemical Engineering, Huaiyin Normal University, No. 111 West Changjiang Road, Huaian, Jiangsu 223300, China

## Abstract

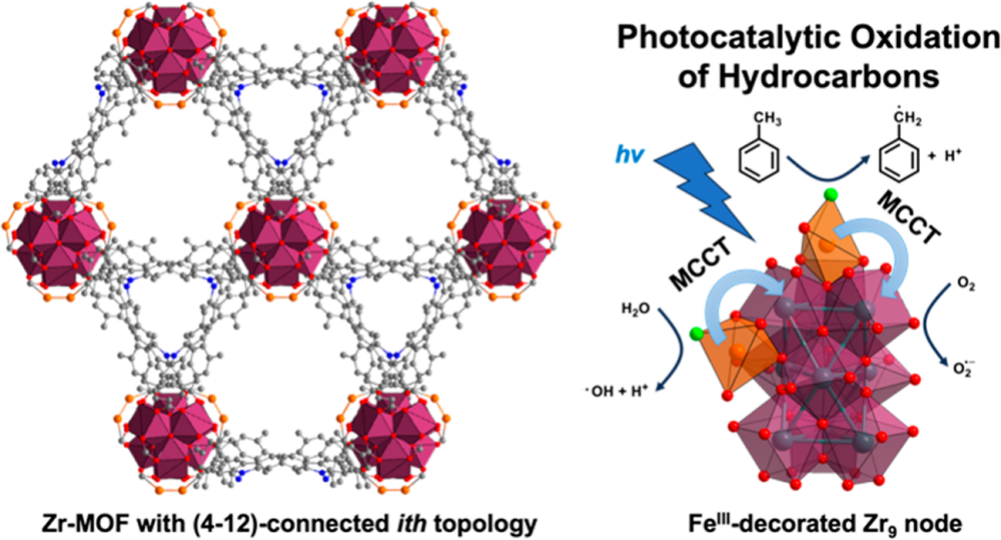

Heterogeneous catalysis plays an indispensable role in chemical production and energy conversion. Incorporation of transition metals into metal oxides and zeolites is a common strategy to fine-tune the activity and selectivity of the resulting solid catalysts, as either the active center or promotor. Studying the underlying mechanism is however challenging. Decorating the metal-oxo clusters with transition metals in metal–organic frameworks (MOFs) via postsynthetic modification offers a rational approach to construct well-defined structural models for better understanding of the reaction mechanism. Therefore, it is important to expand the materials scope beyond the currently widely studied zirconium MOFs consisting of Zr_6_ nodes. In this work, we report the design and synthesis of a new (4,12)-connected Zr-MOF with **ith** topology that consists of rare Zr_9_ nodes. Fe^III^ was further incorporated onto the Zr_9_ nodes of the framework, and the resulting MOF material exhibits significantly enhanced activity and selectivity toward the photocatalytic oxidation of toluene. This work demonstrates a delicate ligand design strategy to control the nuclearity of Zr-oxo clusters, which further dictates the number and binding sites of transition metals and the overall photocatalytic activity toward C–H activation. Our work paves the way for future exploration of the structure–activity study of catalysts using MOFs as the model system.

## Introduction

Heterogeneous catalysis typically involves reusable solid phase catalysts that are readily separated from the reaction mixture, which has laid the foundation for many applications in daily life ranging from fine chemical production to energy conversion.^1^ Typical heterogeneous catalysts are based on solid supports such as metal oxides and zeolites that incorporate active metal species.^2,3^ Indeed, the binding between a metal catalyst and its support significantly affects the selectivity and activity via the coordination mode, geometry, and electronic structures.^4,5^ However, elucidation of the structure–activity relationship in heterogeneous catalysts is a daunting task since it is challenging to identify the active species due to the amorphous nature of many solid supports and inhomogeneous binding sites. Moreover, the tunability of most common solid supports is quite limited, which also hampers the establishment of a systematic variable for the thorough investigation within a series of supports.

Metal–organic frameworks,^6−11^ a class of highly porous crystalline materials that are composed of organic linkers and inorganic nodes, represent an ideal platform to meet the needs as emerging heterogeneous catalysts.^12−16^ The periodic spacing of the metal nodes and ligands, as well as the well-ordered pore structures, enables the systematic study of the structure–activity relationships of the catalytic reactions.^17−23^ In addition, for metal–organic frameworks (MOFs) with the same topology, one can use reticular chemistry to finetune the pore size, an important feature for understanding the structure–activity relationship that is not attenable in other solid supports.^24^ Zr-based MOFs (Zr-MOFs) are an ideal choice of the solid support material due to their outstanding thermal and chemical stability.^25^ The terminal and bridging hydroxyl/aqua groups (−OH/H_2_O) on the Zr-oxo nodes can serve as the binding sites for extraneous metal, and their number and availability are controlled by ligand connectivity and Zr nuclearity. The uniform distribution of the metal species with atomic precision can not only avoid aggregation but also facilitate the structural determination by a variety of spectroscopy methods and single-crystal X-ray diffraction (sc-XRD), further underlining the importance of Zr-MOFs as the ideal model catalyst supports.^26−32^

To date, diverse MOFs featuring Zr_6_,^33−39^ Zr_8_,^40^ and Zr_12_^41^ nodes have been widely studied as porous supports for the facile construction of single-site catalysts in heterogeneous catalysis.^42^ We envision that the design space can be further expanded for Zr-MOFs with other types of nodes with different Zr nuclearity, such as the newly discovered Zr_9_ node.^43−45^ In particular, with the composition of Zr_9_O_9_(OH)_6_(H_2_O)_6_ in (4,12)-connected Zr-MOFs with **ith** topology, the terminal −OH/H_2_O groups, which are absent in the conventional 12-connected Zr_6_ node in the UiO series, offer new opportunities to introduce metal species onto the node. The distinct binding manner of terminal carboxylates with the Zr_9_ node is likely to provide a new local chemical environment that steers the reactivity. Unfortunately, the design insight for the synthesis of (4,12)-connected Zr-MOFs consisting of Zr_9_ nodes, besides a pseudotetrahedral ligand being used for the construction of such MOFs, needs further development.^43^ Herein, we screened several tetrahedral ligands based on tetraphenylmethane and *N*,*N*′-bicarbazole backbone together with synthesis condition optimization and successfully prepared a new (4,12)-connected Zr-MOF with **ith** topology that consists of Zr_9_ nodes, i.e., NPF-520 (NPF = Nebraska porous framework). We further incorporate Fe^III^ ions into NPF-520 by base-promoted coordinative binding to the Zr_9_ nodes, resulting in Fe-doped NPF-520-Fe^III^. With three Fe^III^ ions incorporated in each Zr_9_ node and near-visible absorption of the bicarbazole ligand, NPF-520-Fe^III^ exhibits enhanced activity and selectivity toward the photocatalytic oxidation of hydrocarbons.

## Results and Discussion

### Ligand Steric Tuning

**Figure 1:**
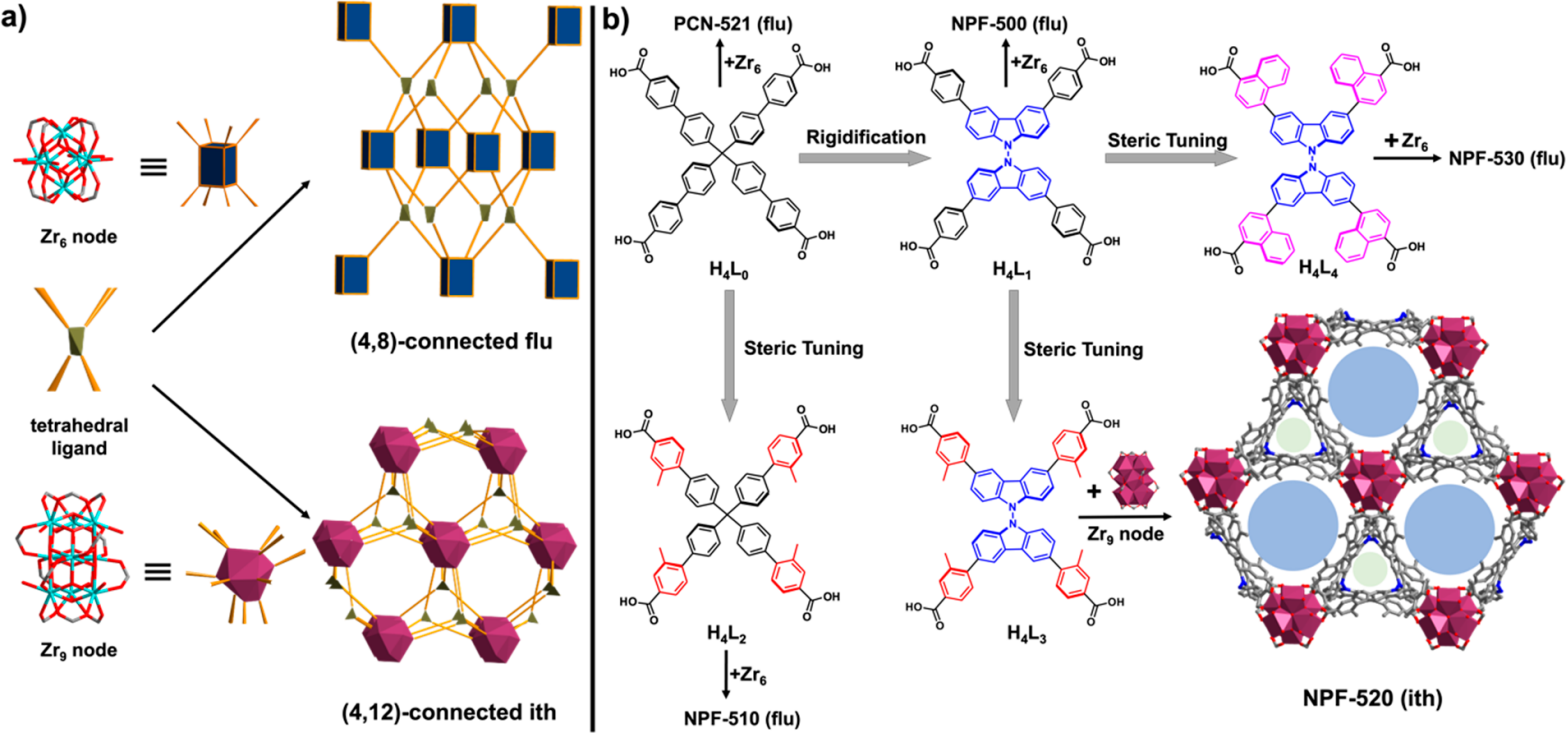
(a) Formation of (4,8)-connected **flu** and (4,12)-connected **ith** topologies from a tetrahedral ligand with Zr_6_ and Zr_9_ nodes, respectively. (b) Sequential rigidification and steric tuning of tetrahedral ligands to form NPF-520 with **ith** topology.

The screening of tetrahedral ligands starts with H_4_**L**_**0**_ (Figure 1), which has been used by Zhou and co-workers to construct a (4,8)-connected Zr-MOF (i.e., PCN-521) with the **flu** topology (Figure 1b).^46^ Previous work by our group showed that rigidification of the four phenyl rings in *N*,*N*′-bicarbazole-based ligand H_4_**L**_**1**_ does not affect the topology of the resulting (4,8)-connected Zr-MOF (i.e., NPF-500, Figure 1b).^47^ We reasoned that further tuning the sterics of terminal benzoates of these two ligands by functionalizing with a methyl group at the 3-position might lead to a proper ligand geometry that facilitates the formation of the targeted (4,12)-Zr-MOF. To our delight, although with the additional methyl groups H_4_**L**_**2**_ (Figure 1b) still results in a (4,8)-connected NPF-510 with the **flu** topology (Table S1), such a strategy proves to be successful for the modification of H_4_**L**_**1**_: with the two additional methyl groups, H_4_**L**_**3**_ forms a Zr-MOF named NPF-520, with the desired **ith** topology that is composed of Zr_9_O_9_(OH)_6_(H_2_O)_6_ nodes (Figure 1b, Table S2). Interestingly, further increase of the steric effect of H_4_**L**_**1**_ by replacing the terminal benzoate with naphthalene carboxylate in ligand H_4_**L**_**4**_ retains the topology of the resulting NPF-530 as **flu** (Figure 1b, Table S3), which underlines the delicate impact of ligand steric and consequent MOF topology. It should be noted here, however, that besides the choice of ligand, other synthetic parameters such as modulator also contribute to the formation of Zr-MOFs with a specific topology. Nevertheless, such steric tuning outlined in this work offers a solid strategy to targeted synthesis of MOFs with specific topological structures.

**Figure 2:**
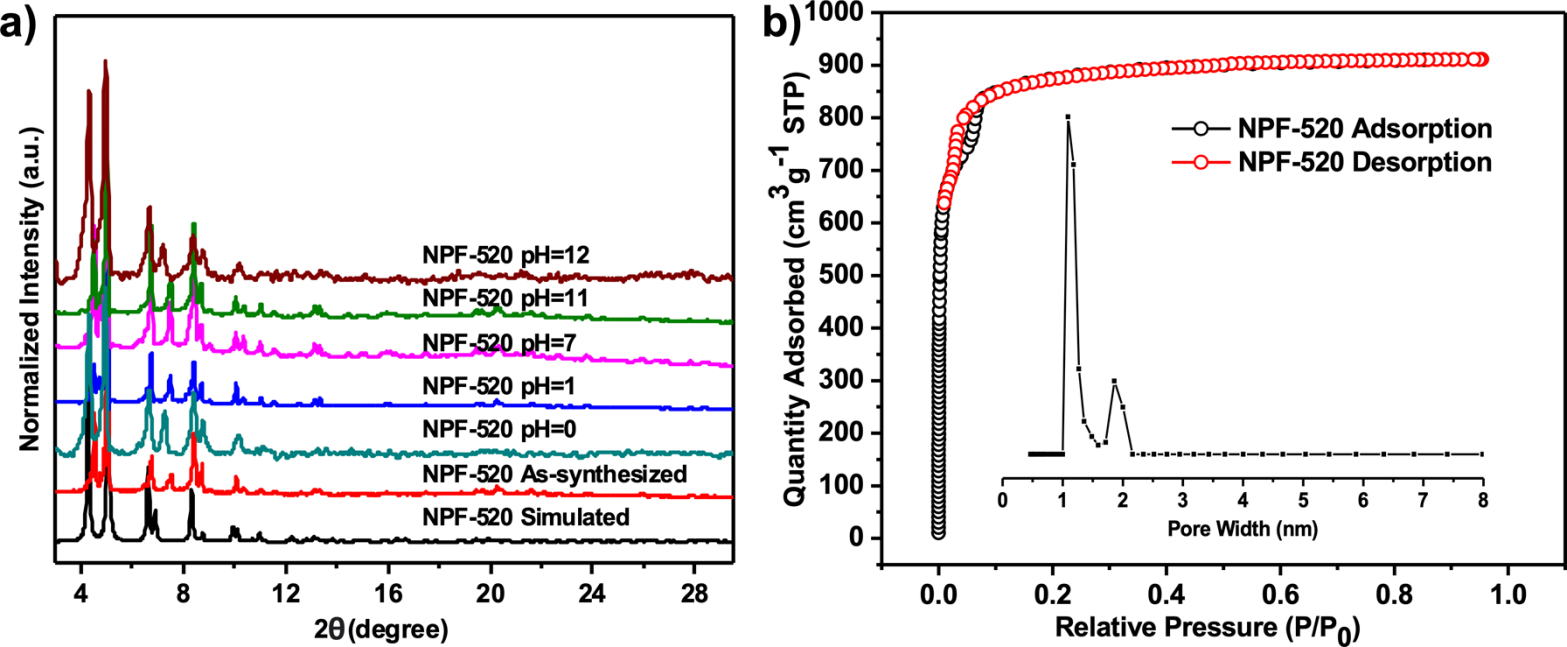
(a) PXRD patterns of NPF-520 after acid, water, and base treatment. (b) N_2_ sorption isotherm at 77 K and pore size distribution of NPF-520.

The synthesis and structural characterization of NPF-510 and NPF-530 are described in the Supporting Information, and here we focus on the discussion of NPF-520. Tetratopic ligand H_4_**L**_**3**_ was synthesized via Suzuki coupling of tetrabromo-*N*,*N*′-bicarbazole and hexyl 3-methyl-4-(4,4,5,5-tetramethyl-1,3,2-dioxaborolan-2-yl)benzoate followed by hydrolysis in a basic medium (see Supporting Information for detailed procedures). Colorless hexagonal crystals of NPF-520 were obtained by solvothermal reaction of ZrCl_4_ and H_4_**L**_**3**_ in the presence of acetic acid as the modulation agent at 120 °C for 48 h (Figure S7). sc-XRD studies at 273 K reveal that NPF-520 crystallizes in the trigonal crystal system, in chiral space group *R*32 with the lattice parameters *a* = *b* = 35.168 Å and *c* = 28.593 Å (Table S2). Close examination of the zirconium cluster reveals two crystallographically distinct Zr atoms (i.e., Zr1 and Zr2) in each asymmetric unit (Figure S8). Zr1 is bridged by eight oxygen atoms, derived from four μ_3_-O^2–^/OH^–^ groups, as well as three **L**_**3**_ ligands and a capping H_2_O molecule. Zr2 is bridged by two oxygen atoms emanating from two **L**_**3**_ ligands and seven μ_3_-O^2–^/OH^–^ groups. Grouped together, six Zr1 and three Zr2 atoms are bound by eight μ_3_-O^2–^/OH^–^ groups, forming the rare Zr_9_ nodes. Topologically, each Zr_9_ cluster can be described as the face-sharing of two Zr_6_ clusters that links 12 tetrahedral ligands (Figure S8), and each tetrahedral ligand is bridged by four Zr_9_ clusters to give an extremely rare (4,12)-connected **ith** net (Figure 1b).^43,48^ Such interconnection between the ligand and Zr_9_ node results in a 3D framework with a charge-balanced formula of Zr_9_O_9_(OH)_6_(H_2_O)_6_(**L**_**4**_)_3_. The resulting 3D framework contains two types of triangular 1D channels with diameters of 1.2 and 0.4 nm along the *c*-axis (Figure 1b). PLATON calculations indicate the presence of 66.7% of void space accessible for guest molecules.^49^ Powder X-ray diffraction (PXRD) patterns of NPF-520 confirm the bulk phase purity of the as-synthesized sample when compared with the simulated patterns from its corresponding single-crystal structure (Figure 2a).

### Stability and Porosity of NPF-520

The thermal stability of NPF-520 was studied by thermogravimetric analysis (TGA) under N_2_. The initial weight loss in the temperature range of 150 to 250 °C is attributed to the removal of the solvent molecules, and NPF-520 exhibits excellent thermal stability exemplified by the thermal decomposition around 540 °C (Figure S12). In addition, the chemical stability of NPF-520 was examined by treating MOFs in H_2_O, basic (pH = 11 and 12), and acidic (pH = 1 and 0) conditions. The PXRD patterns after 24 h remained intact, suggesting good stability with no phase transition or framework collapse (Figure 2a). After activation using supercritical CO_2_ exchange, the permanent porosity of NPF-520 was measured by N_2_ adsorption isotherms at 77 K (Figure 2b). NPF-520 exhibits a typical type I isotherm with a saturated N_2_ uptake of 915 cm^3^ g^–1^. The Brunauer–Emmett–Teller surface area (SA_BET_) was determined to be 3463 m^2^ g^–1^, consistent with the calculated accessible surface area of 3358 m^2^ g^–1^.

### Postsynthetic Incorporation of Fe

Many studies of transition-metal-doped Zr-MOFs have been focused on chemical catalysis in gas and solution phases, and recently photocatalytic applications of these materials have also emerged.^50−53^ One interesting strategy reported by Jiang and co-workers involves the incorporation of Fe^III^ onto the Zr-oxo clusters of UiO-66, which not only creates catalytic active center but also shifts the light absorbance that is attributed to metal-to-cluster or metal-to-metal charge transfer (MCCT or MMCT) from Fe^III^ to the Zr_6_ node.^54^ It was proposed that the photoinduced charge separation originated from MCCT^55−57^ promotes the oxidation of H_2_O to hydroxyl radical (^•^OH), which subsequently activates C–H bonds of toluene in the presence of O_2_ and ultimately the formation of benzoic acid. We hypothesize that the different local chemical environment around the Fe^III^-decorated Zr_9_ node in NPF-520-Fe^III^ and/or a photoactive bicarbazole-based ligand might induce a different photocatalytic activity or selectivity toward C–H activation.

In the past, the incorporation of Fe^III^ into Zr-MOFs has been realized by microwave-^54^ and solvent-assisted^58^ synthesis, by which ∼1.2 Fe and up to 2.2 Fe atoms per Zr_6_ node were introduced on UiO-66 and NU-1000, respectively. Here, we adopted a deprotonation-assisted metalation strategy developed by Lin and co-workers.^59^ Briefly, NPF-520 was first deprotonated with trimethylsilylmethyllithium followed by reaction with anhydrous FeCl_3_ to afford the Fe-modified MOF NPF-520-Fe^III^. ICP-OES analysis indicates that 3.1 Fe atoms were incorporated onto each Zr_9_ node. PXRD patterns show that the framework structure of NPF-520 is retained after metalation (Figure S20a). Installation of Fe centers reduced SA_BET_ from 3463 m^2^/g to 2511 m^2^/g for NPF-520-Fe^III^, and the pore size also decreased slightly from 1.9/1.1 nm to 1.6/0.9 nm (Figure S20b). The hexagonal crystal shape of NPF-520 was retained with no additional particles forming, as seen by SEM images (Figure S20c,d). Energy dispersive X-ray (EDX) mapping analysis of NPF-520-Fe^III^ also shows a similar distribution of Fe and Zr throughout the crystals (Figure S21), consistent with the even distribution of Fe on the Zr_9_ clusters. The ^1^H NMR spectrum of NPF-520-Fe^III^ digested with sulfuric acid in D_2_O confirmed that the linker was intact after the chemical modification (Figure S22).

**Figure 3:**
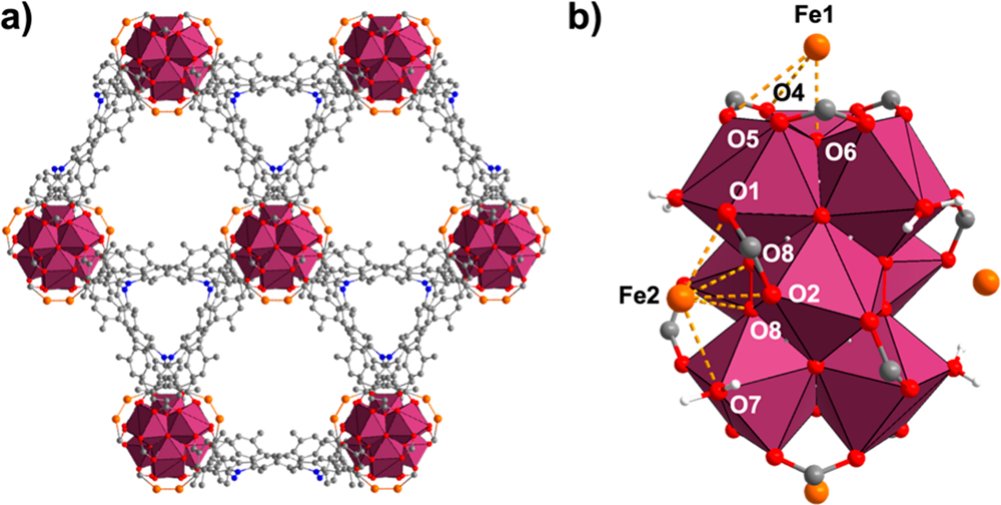
(a) Single-crystal structure of NPF-520-Fe^III^ viewed down the *c*-axis. (b) Side view of the Zr_9_ node showing the two crystallographically distinct Fe sites.

Single-crystal XRD analysis was conducted to obtain structural insights into the incorporated Fe^III^ species in NPF-520-Fe^III^ (Figure 3a and Table S4). After refinement of the parent framework structure, the residual electron density was calculated to assist in the identification of the location and occupancy of Fe. There are two crystallographically distinct Fe sites (i.e., Fe1 and Fe2) in each asymmetric unit (Figure 3b). Fe1 is located inside the two corners of the trigonal bipyramidal cage (Figure S23) and coordinated to the Zr_9_ node through μ_3_-bridging oxygen (O6, 2.63 Å) and carboxylate oxygens (O4/O5, 3.15 and 3.19 Å). Fe2 sits in the triangular pore and coordinates to the Zr_9_ node through terminal oxygen (O7, 2.84 Å), carboxylate oxygens (O1/O2, 2.56 and 2.45 Å), and μ_3_-bridging oxygens (O8, 3.02 and 2.92 Å). The total occupancies of the Fe sites are 1.15 (Fe1) and 1.66 (Fe2) per Zr_9_ node, which corresponds to a total iron content of 2.81 Fe/Zr_9_, consistent with the ICP-OES data (3.1 Fe/Zr_9_). The location of Cl^–^ could not be determined, likely due to severe disorder and low occupancy. The relatively long Fe–O distances (2.5–3.2 Å) suggest a weak interaction between Fe and the Zr node at the solvated state during which diffraction data were collected. Thus, although sc-XRD is helpful to reveal the general location and occupancy, the local coordination of Fe needs further investigation.

**Figure 4:**
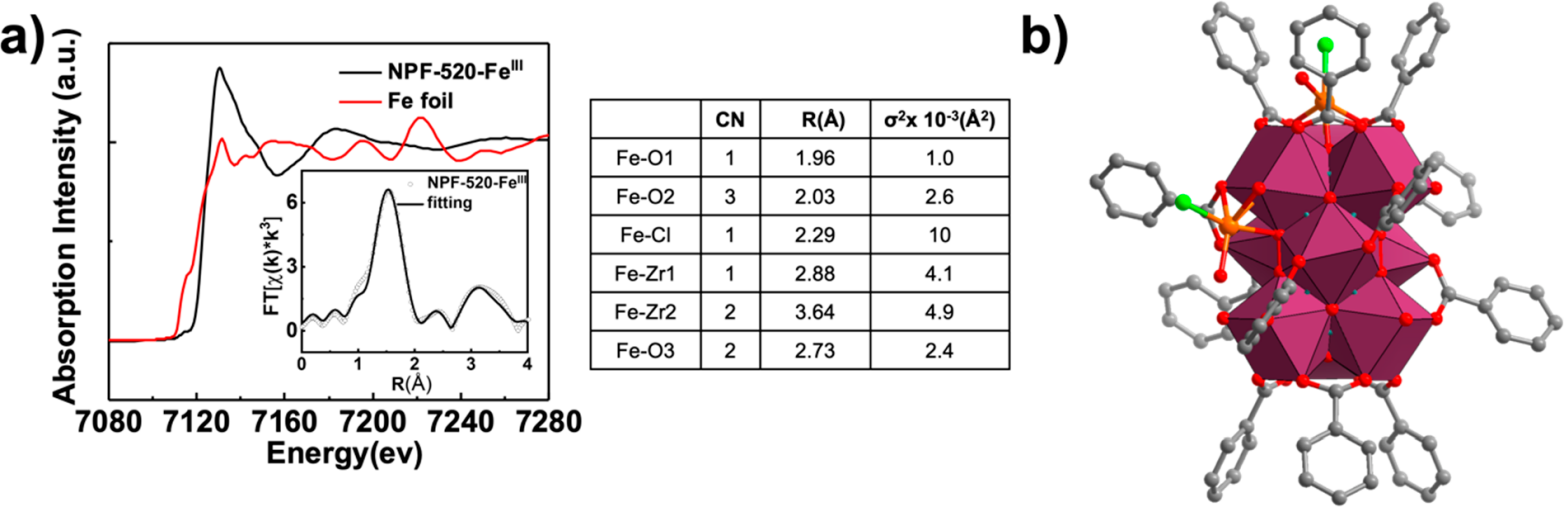
(a) Fe K-edge XANES of NPF-520-Fe^III^ and Fe foil and fitting parameters (inset: EXAFS spectrum and the fit). (b) Structural model of the Fe^III^-decorated Zr_9_ node used for EXAFS fitting.

Next, X-ray absorption spectroscopy (XAS) was performed to gain direct insights into the local electronic structure at the Fe center in NPF-520-Fe^III^. The X-ray absorption near edge (XANES) spectrum of the Fe foil reference exhibits the main feature at 7112 eV, corresponding to the Fe 1s–4p transition (Figure 4a). In contrast, an additional weak pre-edge feature corresponding to the quadrupole allowed 1s–3d transition was observed in the spectrum of NPF-520-Fe^III^, indicating the non-centrosymmetric geometry of the Fe center (Figure 4a). To quantitatively examine the local coordination environment, extended X-ray absorption fine structure (EXAFS) spectra at the Fe K-edge were fit using the Demeter X-ray absorption analysis package. The R-space spectrum with the best fit is shown in Figure 4a with fitting parameters. Compared to Fe reference foil (Figure S24), no prominent Fe–Fe bond was observed in NPF-520-Fe^III^, consistent with the fact that the multiple Fe2 sites are the result of symmetry generation instead of a real close Fe–Fe contact. The first shell was dominated by an Fe–O/Cl bond with an average coordination number of five. From the best fitting results, the bond distances of Fe–O and Fe–Cl were determined to be between 1.96 and 2.29 Å and were consistent with a structural model shown in Figure 4b, in which Fe1 is coordinated with two carboxylate oxygens, one μ_3_-bridging oxygen, one chloride, and one oxygen from water or hydroxide, and Fe2 is coordinated with one carboxylate oxygen, one terminal oxygen, one μ_3_-bridging oxygen, one chloride, and one oxygen from water or hydroxide. X-ray photoelectron spectroscopy (XPS) was further used to study the chemical states of Zr and Fe in NPF-520 and NPF-520-Fe^III^. The binding energy of 711.2 eV of Fe 2p_3/2_ confirms the Fe^III^ state in NPF-520-Fe^III^ (Figure S25a). Importantly, due to the stronger electron-withdrawing effect of Fe^III^, the peak of Zr 3d_5/2_ shifts from 181.78 eV in NPF-520 to 182.10 eV in NPF-520-Fe^III^ (Figure S25b,c), consistent with the formation of Zr^IV^-O-Fe^III^ in the latter.

The electronic structure of NPF-520 before and after the incorporation of Fe^III^ was also studied. UV–vis spectra show a red shift and enhanced visible light absorption after the incorporation of Fe^III^, consistent with its light-yellow color (Figure S26). The positive slopes of the linear Mott–Schottky plots of NPF-520 and NPF-520-Fe^III^ at different frequencies indicate the nature of the n-type semiconductor (Figure S27). The LUMO energy levels were determined from the intersection with the values of −0.77 and −0.42 V vs Ag/AgCl (i.e., −0.58 and −0.23 V vs NHE) for NPF-520 and NPF-520-Fe^III^, respectively.^50^ Combined with the band gaps estimated by Tauc plots (Figure S26), the energy diagrams were determined (Figure S28), which suggests that the incorporation of Fe^III^ lowers the LUMO energy level while it raises the HOMO energy level, resulting in the decrease of the band gap from 4.20 eV to 3.19 eV, a value comparable to that of the previously reported Fe-UiO-66 (i.e., 3.02 eV).^54^ From the thermodynamic perspective, similar to Fe-UiO-66, NPF-520-Fe^III^ has the capability for water oxidation (*E*_•OH/OH–_ = 1.89 V vs NHE; *E*_O2/H2O_ = 1.23 V vs NHE) as well as O_2_ reduction (*E*_O2/O2•–_ = −0.16 V).^60^

### Photocatalytic Toluene Oxidation

In view of the appropriate energy levels of NPF-520-Fe^III^, we next used the toluene oxidation reaction to evaluate its photocatalytic performance in C–H activation. Under visible light irradiation from a blue LED photoreactor (λ = 395 nm), toluene oxidation was first carried out in the presence of 5 mol % photocatalyst and O_2_ (1 atm). To our delight, the reaction gives 100% conversion in 8 h with nearly exclusive selectivity to benzaldehyde (entry 1 and Table 1), which is consistent with the fact that the absence of water eliminates the production of a hydroxyl radical required for the complete oxidation to benzoic acid. Continuing to run the reaction for another 16 h still does not produce any detectable benzoic acid. Hot filtration experiments demonstrate that oxidation catalysis occurs heterogeneously (Figure S29). However, upon the introduction of 20 μL of water into the reaction mixture, benzoic acid started to emerge (41%, entry 3, Table 1, Figure S30). Complete benzoic acid selectivity was achieved within 6 h by increasing the amount of water to 100 μL (entry 4, Table 1 and Figure S30). As expected, the reaction does not proceed in the absence of O_2_ (entries 5 and 6, Table 1), and unmodified NPF-520 and FeCl_3_ show no catalytic activity under the same reaction conditions (entries 7–11, Table 1), confirming the photoactivity originating from MCCT due to Fe incorporation. NPF-520-Fe^III^ exhibits good stability, suggested by similar PXRD patterns after catalysis (Figure S31). ICP-OES indicates negligible iron leaching (<1%) after the first run, and the iron content remains stable after the next four repetitions at room temperature (Table S5). The recyclability of the MOFs is also well maintained within five consecutive runs (Figure S32).

**Table 1:**
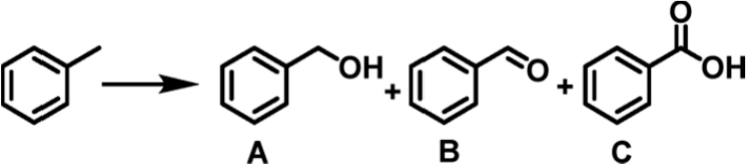
Toluene Oxidation under Different Conditions^a^

entry	catalyst	H_2_O/μL	*t*/h	conversion^b^	selectivity^b^ (A/B/C)
1	NPF-520-Fe^III^	0	8	100	0/100/0
2	NPF-520-Fe^III^	0	24	100	0/100/0
3	NPF-520-Fe^III^	20	8	41	0/94/6
4	NPF-520-Fe^III^	100	6	100	0/0/100
5^c^	NPF-520-Fe^III^	0	8	0	
6^c^	NPF-520-Fe^III^	100	6	0	
7	NPF-520	0	12	0	
8	NPF-520	100	12	0	
9	FeCl_3_	0	12	0	
10	FeCl_3_	100	12	0	
11	UiO-66-Fe^III^	0	12	0	
12	UiO-69-Fe^III^	0	12	4	0/100/0
13	UiO-66-Fe^III^	100	12	6	0/36/64
14	UiO-69-Fe^III^	100	12	12	0/18/82

a Reaction conditions: 5 μL of toluene, 1 mL of MeCN, 5 mol % catalyst (based on Fe^III^), 395 nm blue LED photoreactor, 1 atm O_2_.

b Determined by GC/GC-MS.

c Without O_2_.

To further benchmark the catalytic activity of NPF-520-Fe^III^, we synthesized two Fe^III^-doped Zr-MOFs, UiO-66-Fe^III^ (1.1 Fe per Zr_6_ node) and UiO-69-Fe^III^ (1.2 Fe per Zr_6_ node), as the comparison adopting a literature synthesis procedure (see Supporting Information for details).^59^ Both UiO materials exhibit inferior activities compared to NPF-520-Fe^III^ under similar reaction conditions (entries 11–14, Table 1). Although the larger channel size of UiO-69-Fe^III^ is likely responsible for its slightly higher activity compared with UiO-66-Fe^III^, the overall significantly lower activity of UiO materials suggests an enhanced energy utilization efficiency in NPF-520-Fe^III^. On one hand, the presence of near-visible absorption of the bicarbazole ligand in NPF-520 increases light harvesting, which likely undergoes energy transfer to the MMCT process. This is corroborated by the decreased fluorescence of NPF-520 upon Fe^III^ doping (Figure S33). On the other hand, compared to the Zr_6_ nodes doped with a single Fe^III^ ion, a more efficient charge separation process might occur in Zr_9_ nodes doped with ∼3 Fe^III^ ions in NPF-520-Fe^III^. Further investigation is required to elucidate the dominant contribution; nevertheless, it is clear that modification of the tetrahedral ligand not only facilitates the formation of the Zr_9_ nodes but also enhances the light harvesting, contributing to both the enhanced activity and selectivity toward the photocatalytic oxidation of hydrocarbons.

### Photocatalytic Mechanism

**Figure 5:**
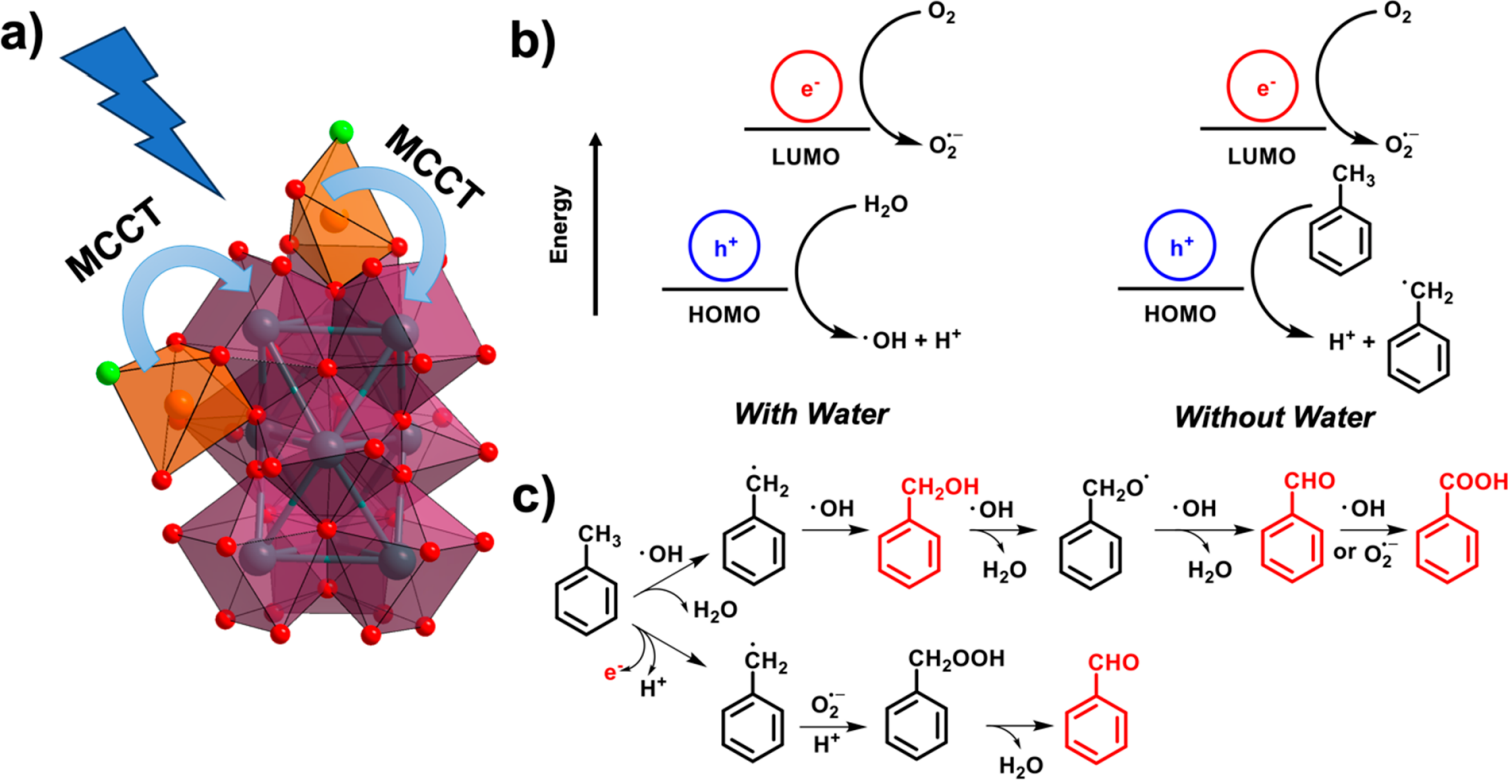
(a) Schematic illustration of the Fe^III^ to Zr-oxo cluster charge transfer process in the Fe^III^-decorated Zr_9_ node (Zr: violet, O: red, Fe: orange; Cl: green). (b) Generation of reaction intermediates in the presence of water and under anhydrous conditions. (c) Proposed mechanism for the photocatalytic oxidation of toluene.

Our work demonstrates that, depending on the availability of water in the reaction system, the MCCT process can lead to a different product selectivity, which we attribute to the different reactive oxygen species (ROS) (i.e., hydroxyl radical ^•^OH and superoxide O_2_^•–^) generated under the different conditions. We propose the mechanism for the photocatalytic oxidation of toluene as follows (Figure 5). In the presence of water, the photoexcited hole is transferred to H_2_O, resulting in the formation of the hydroxyl radical ^•^OH and proton. The photoexcited electron then reacts with the O_2_ to form O_2_^•–^. From there, the generated ROS, ^•^OH and O_2_^•–^, can react with toluene and its partially oxidized derivatives to form benzyl alcohol, benzaldehyde, and benzoic acid. In the absence of water, the photoexcited hole can only react with toluene to form the toluene radical and H^+^, which then combines with O_2_^•–^ to form benzaldehyde. The superoxide does not further react with benzaldehyde to form benzoic acid, resulting in selectivity for the aldehyde product, which is observed in all cases when the photooxidation reaction is conducted under anhydrous conditions.

Several sets of experiments were conducted to provide further support for the proposed mechanism. The presence of these produced ROS can be detected using certain agents, such as terephthalic acid for ^•^OH and luminol for O_2_^•–^.^61^ As expected, under anhydrous conditions, no ^•^OH was produced, as no fluorescence signal was observed around 430 nm due to the oxidation of terephthalic acid (Figure S34). This is consistent with the proposed reaction mechanism where water reacts with the photogenerated hole to produce ^•^OH. However, the luminol-amplified chemiluminescence test indicates that O_2_^•–^ is produced regardless of the presence of water (Figure S35), confirming that O_2_ gas is indeed the electron acceptor. Moreover, quenchers such as 2-methylfuran (for singlet oxygen), 1,4-diazabicyclo[2.2.2]octane (DABCO, for singlet oxygen), and 2,2,6,6-tetramethylpiperidine 1-oxyl (TEMPO, for all radicals) were also used to help identify ROS involved in the reaction.^61^ According to Table S6, the addition of 2-methylfuran and DABCO did not affect the photocatalytic reaction at all, suggesting that singlet oxygen is not involved in the oxidation reaction. On the other hand, TEMPO completely quenched the reaction, highlighting the importance of radical species in the photocatalysis. Besides the radical quenchers, sacrificial electron or hole donors were also added to the reaction mixture to confirm if the oxidation was hole or electron driven. While the sacrificial electron donor triethylamine completely quenched the reaction, the reaction proceeded in the presence of (NH_4_)_2_Ce(NO_3_)_6_, suggesting that the photooxidation is hole-driven (Table S6). Finally, hydrogen peroxide (H_2_O_2_) affords only minimal conversion, suggesting the Fenton reaction is not the dominant reaction driving the toluene oxidation (Table S6).

The kinetic isotope effect (KIE) was studied to determine the rate-limiting step in the photo-oxidation of toluene (Figure S36). Although a minimal KIE (1.06) was observed when D_2_O was used instead of H_2_O, switching toluene to toluene-*d*_8_ demonstrated the largest KIE (i.e., 3.23 in the presence of water and 6.67 in the absence of water), indicating that C–H bond activation is the rate-limiting step under both reaction conditions. In the absence of water, hole-to-toluene oxidation instead occurs to produce the toluene radical, which then reacts with ROS such as superoxide to form benzaldehyde. Under anhydrous conditions, the rate-limiting effect of toluene is even greater than with water present. Finally, although O^18^_2_ shows a KIE of more than 1 for both reactions (Figure S36), indicating that the breaking of the O–O bond is crucial to the reaction, it is not the predominant rate-limiting step.

## Conclusions

In summary, we have demonstrated a rational strategy to construct a Zr-MOF consisting of nonanuclear nodes that undergoes a postsynthetic modification to afford a highly active photocatalyst for aerobic C–H activation. Using ligand rigidification and steric tuning, we successfully modified an *N*,*N*′-bicarbazole-based tetrahedral ligand and synthesized a highly stable Zr-MOF named NPF-520 that features the (4,12)-connected **ith** topology. The unique metal binding sites on the Zr_9_ node in NPF-520 facilitate the addition of up to three Fe^III^ atoms to the nonanuclear Zr-oxo clusters. The resulting NPF-520-Fe^III^ exhibits significantly enhanced photocatalytic activity toward toluene oxidation. Moreover, the reaction selectivity can be controlled by varying the water content to afford exclusively either aldehyde or benzoic acid as the oxidation product via an MCCT process that likely involves the enhanced light harvesting via the near-visible absorption of ligand. This work highlights the importance of the metal-binding mode of a metal-oxo cluster to modulate the catalytic activity of MOFs for heterogeneous catalysis and paves the road for building model systems to understand the structure–activity relationship for broader application avenues of MOFs for both chemical and photochemical catalysis.

## Supporting Information Available

Synthesis, experimental data and procedures, single-crystal X-ray diffraction, TGA, PXRD, N_2_ sorption isotherms, postsynthetic modification, and photocatalysis data (PDF)
