# Supplementary material for: Postsynthetic Modification of the Nonanuclear Node in a Zirconium Metal–Organic Framework for Photocatalytic Oxidation of Hydrocarbons
Source: J Am Chem Soc. 2023 Oct 25;145(44):24052–60. doi: 10.1021/jacs.3c07237 (PMC10636760; doi:10.1021/jacs.3c07237)
Supplement: Supplementary file 1 — ja3c07237_si_001.pdf [file ja3c07237_si_001.pdf]

Supporting Information

Postsynthetic Modification of Nonanuclear Node in Zirconium Metal-Organic Framework for Photocatalytic Oxidation of Hydrocarbons

Rebecca Shu Hui Khoo,[1,‡] Christian Fiankor,[2,‡] Sizhuo Yang,[1] Wenhui Hu,[3] Chongqing Yang,[1] Jingzhi Lu,[2] Martha D. Morton,[2] Xu Zhang,[4] Yi Liu,[1] Jier Huang,[3] and Jian Zhang[1,2]

[1]The Molecular Foundry, Lawrence Berkeley National Laboratory, Berkeley, California 94720 United States

[2]Department of Chemistry, University of Nebraska-Lincoln, Lincoln, Nebraska, 68588 United States

[3]Department of Chemistry, Marquette University, Milwaukee, Wisconsin, 53201 United States

[4]School of Chemistry and Chemical Engineering, Huaiyin Normal University, Jiangsu Engineering Laboratory for Environment Functional Materials, Jiangsu Collaborative Innovation Center of Regional Modern Agriculture & Environmental Protection, No.111 West Changjiang Road, Huaian, Jiangsu 223300 China

[‡]These authors contributed equally to this work

Table of Contents

**Section S-1**	Materials and Methods	S3
**Section S-2**	Synthesis and Characterization of Organic Ligands	S5
**Section S-3**	MOF Synthesis	S14
**Section S-4**	Optical Microscopic Images	S16
**Section S-5**	Crystallographic Data and Structural Representation	S17
**Section S-6**	Thermogravimetric Analysis	S25
**Section S-7**	Powder X-ray Diffraction	S27
**Section S-8**	N_2 Sorption Isotherms	S29
**Section S-9**	Characterization of NPF-520-Fe^{III}	S30
**Section S-10**	Photocatalysis	S34
**Section S-11**	References	S40

Section S-1 Materials and Methods

All solvents and reagents were purchased from commercial sources and, unless otherwise noted, used without further purification. ^{1}H NMR, and ^{13}C NMR was performed on a 150 MHz, 400 MHz, 600 MHz Bruker FT-NMR spectrometer. Mass spectra (MS) were performed on a Waters Q-TOF I mass spectrometer. Thermogravimetric analysis (TGA) was performed on a TA Instruments Discovery 550 thermogravimetric analyzer, heated from 30°C to 800°C at a rate of 8°C/minute under N_2 atmosphere. Powder X-ray diffraction (PXRD) performed a PANalytical Empyrean diffractometer with a PIXcel 3D detector. The copper target X-ray tube was set to 45 kV and 40 mA. Photocatalytic reactions were conducted using a Penn PhD Photoreactor M2 (Z744035, Sigma Aldrich) equipped with 395 nm LED light sources.

Gas Sorption Measurements. Gas adsorption isotherms were performed on the surface area analyzer, Micromeretics ASAP-2020. N_2 gas adsorption isotherms were measured at 77 K using a liquid N_2 bath.

Scanning Electron Microscope. SEM images and EDS data were collected on a tabletop Phenom ProX equipped with the Element Identification (EID) software package and a specially designed and fully integrated Energy Dispersive Spectrometer (EDS).

Inductively Coupled Plasma-Optical Emission Spectrometry. ICP-OES, performed on a Varian ICP-OES 720 Series, was used to quantify the ratio of the MOF metal and the grafted metal. Samples were digested in piranha overnight with stirring and diluted with 2 wt% HNO_3 before ICP measurement. 1000 ppm zirconium and iron standard solutions (Sigma Aldrich) was used to prepare diluted standards with metal concentrations ranging from 0.1 to 10 ppm.

Fourier transform infrared (FTIR) Spectra. FTIR spectra were recorded on the Nicolet iS50 FT-IR system (Thermo Fisher, USA) using a diamond ATR crystal.

UV-visible Spectroscopy. UV-visible diffuse reflectance data were taken using a Cary 5000 spectrometer with an internal diffuse reflectance accessory.

X-ray absorption (XAS). XAS spectra were measured at the beamline 12BM-B at the Advanced Photon Source in Argonne National Laboratory. The XAS spectra were collected under room temperature with fluorescence mode. The detector was based on 13-element

germanium. One ion chamber is placed before the sample and used as the incident X-ray flux reference signal. There are two ion chambers (second and third chambers) after the sample.

Photoluminescence. A Quantaurus-QY Plus UV-NIR absolute PL quantum yield spectrometer was used to measure the fluorescence spectra. Equivalent 100 µL volumes of sample were removed from the ongoing reaction using a syringe, then diluted to 1 mL and filtered through a syringe filter before measurement.

X-ray Photoelectron Spectroscopy. A Thermo-Fisher K-Alpha Plus XPS with a monochromatic Al X-Ray source (1.486 eV), energy resolution and spatial resolution of 0.7 eV and 30 mm respectively was used to obtain the quantitative chemical analysis of the MOF surfaces.

Activation procedures for N_2 sorption measurements. As-synthesized crystals of NPF-500 series were exchanged with fresh DMF three times every 12 h and heated to 95°C for 10 h. DMF soaked crystals were subsequently exchanged with ethanol three times every 12 h. The samples were activated using $scCO_2$ treatment on a Samdri®-PVT-3D supercritical CO_2 dryer and transferred to a sorption tube for N_2 uptake experiments.

Section S-2 Synthesis and Characterization of Organic Ligands

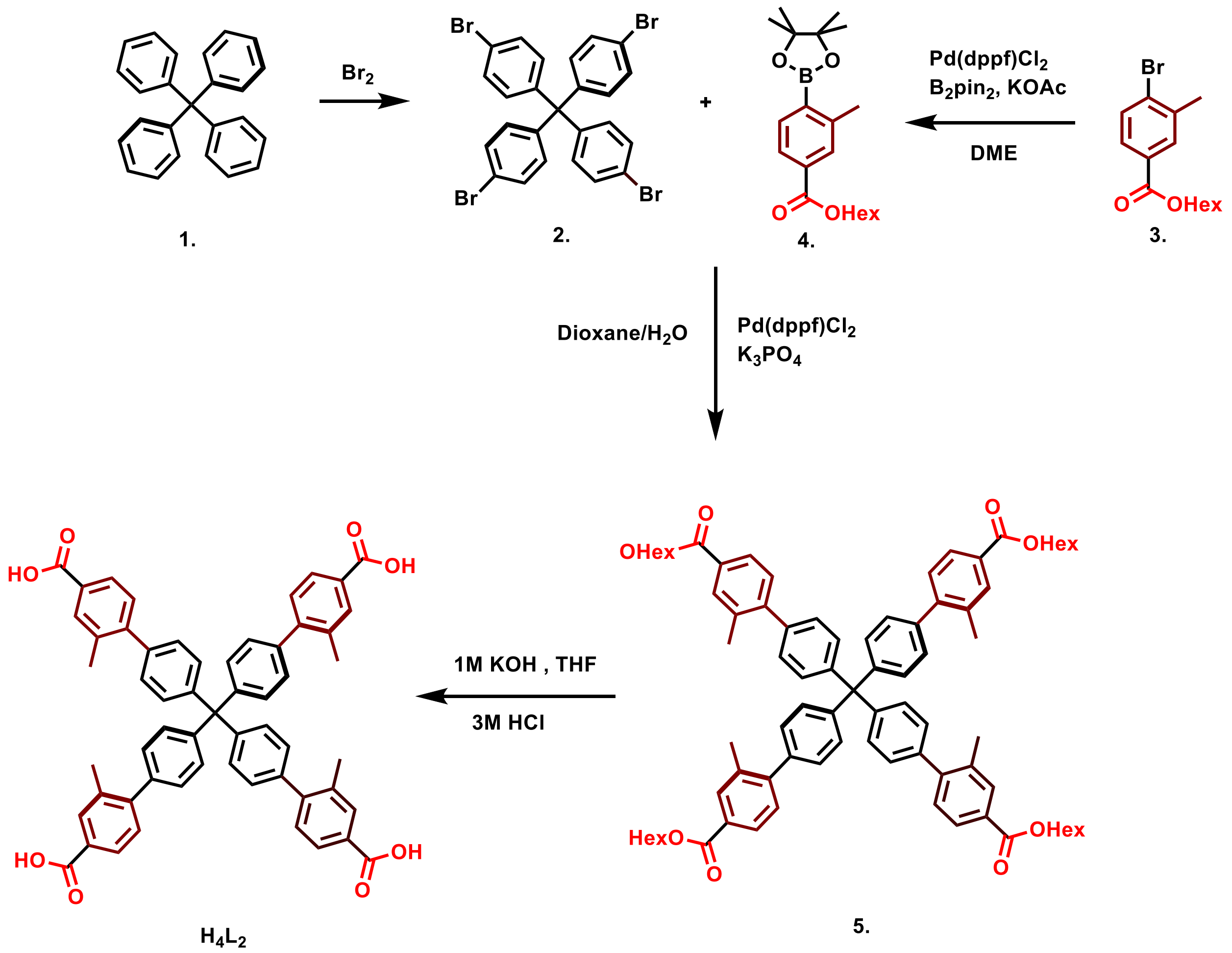

Scheme S1. Synthesis route leading to H_4**L_2**

Tetraphenylmethane (1):

Synthesized according to literature without modification.[1]

Tetrakis(4-bromophenyl)methane (2):

Synthesized according to literature without modification.[1]

Hexyl 4-bromo-3-methylbenzoate (3):

Synthesized according to literature,[2] with the exception that 4-bromo-3-methylbenzoic acid was used as the starting material.

^{1}H NMR (400 MHz, $CDCl_3$) 7.92 (d, J = 1.8 Hz, 1H), 7.77 - 7.68 (m, 1H), 7.61 (d, J = 8.3 Hz, 1H), 4.32 (t, J = 6.7 Hz, 2H), 2.47 (s, 3H), 1.84-1.69 (m, 2H), 1.36 (ddd, J = 8.6, 6.1, 3.3 Hz, 9H), 0.98 - 0.85 (m, 4H).

Hexyl 3-methyl-4-(4,4,5,5-tetramethyl-1,3,2-dioxaborolan-2-yl)benzoate (4):

Synthesized according to literature,[2] with the exception that hexyl 4-bromo-3-methylbenzoate was used as the starting material.

^{1}H NMR (400 MHz, $CDCl_3$) 7.86-7.76 (m, 3H), 4.33 (t, *J* = 6.7 Hz, 2H), 2.60 (s, 3H), 1.84-1.73 (m, 2H), 1.51-1.32 (m, 18H), 0.92 (dd, *J* = 9.7, 4.5 Hz, 3H).

4',4''',4''''',4'''''''-methanetetrayltetrakis(2-methyl-[1,1'-biphenyl]-4-carboxylic acid) (H_4L_2):

Compound 2 (2.1 g, 3.30 mmol), K_3PO_4 (8.4 g, 39.6 mmol), compound 4 (4.29 g, 12.9 mmol) and Pd(dppf)Cl_2 (0.241 g, 0.33 mmol) were charged in a 250 mL 2-neck round bottom flask equipped with a magnetic stir bar. Dioxane (70 mL) and water (10 mL) was added, and the mixture was degassed by sparring with argon for 30 min. The flask was capped and heated to 98°C under inert atmosphere for 70 h. After cooling down to room temperature, the reaction mixture was extracted into chloroform (150 mL × 2) and washed with brine (100 mL). The organic fractions were collected, dried with $MgSO_4$, concentrated under reduced pressure. The white solid was redissolved in DCM, reprecipitated with MeOH and filtered. The solid was transferred to a 500 mL round bottom flask and suspended in 100 mL of THF to which 100 mL of 8 M KOH and 100 mL of MeOH were added. The resulting suspension was stirred under reflux for 48 h. After cooling down to room temperature, the organic solvent was removed in vacuo. The aqueous phase was reprecipitated with 3 M HCl until pH = 2 was reached. The precipitate was filtered, washed with 200 mL of H_2O and dried under vacuum to give H_4L_2 as a colorless solid (1.2 g, yield: 87%).

^{1}H NMR (400 MHz, DMSO) δ 12.87 (s, 4H), 7.88 (d, *J* = 1.3 Hz, 3H), 7.86 – 7.76 (m, 3H), 7.51 – 7.30 (m, 16H), 2.33 (s, 9H).

^{13}C NMR (150 MHz, DMSO, ppm) δ 167.4, 145.1, 138.3, 135.4, 131.4, 130.6, 130.1, 129.7, 128.7, 127.1, 20.4

ESI MS found $[M+H^+]$ m/z= 857.9334, calc $[M+H^+]$ m/z: 857.9729

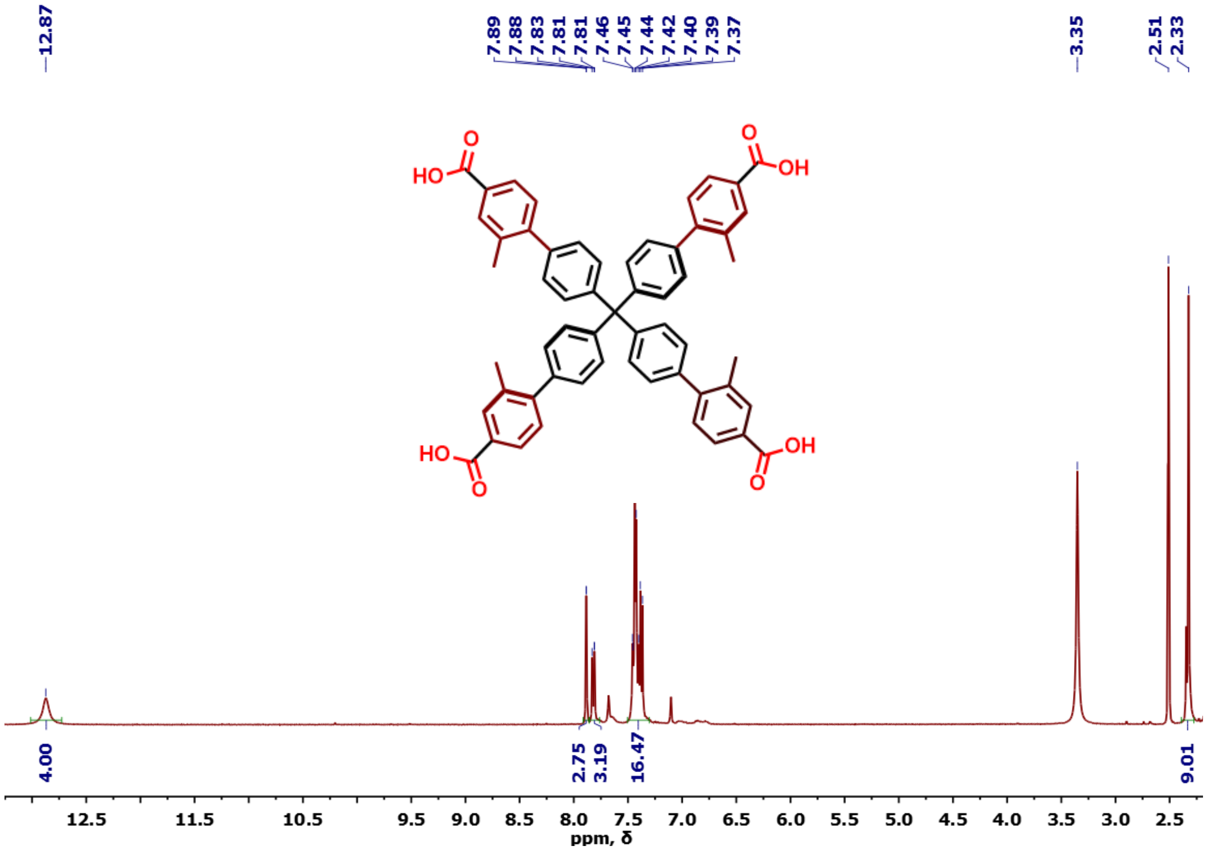

Figure S1. ^{1}H NMR spectrum of H_4**L_2**

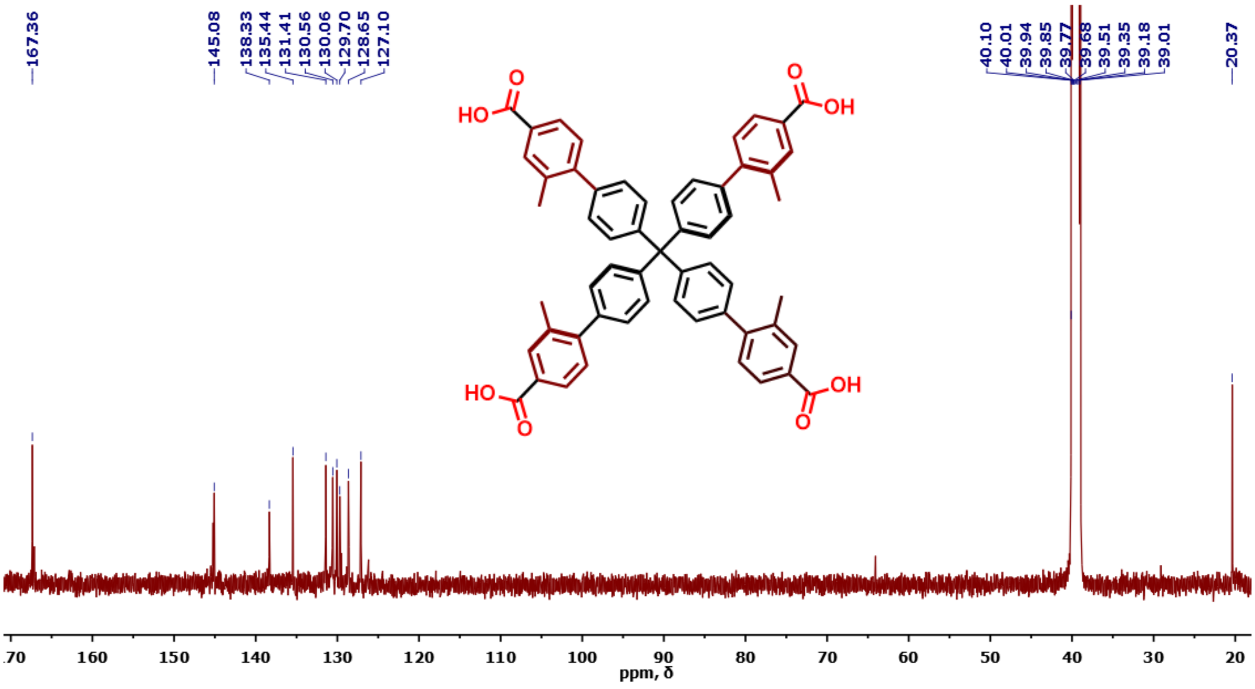

Figure S2. ^{13}C NMR spectrum of H_4**L_2**

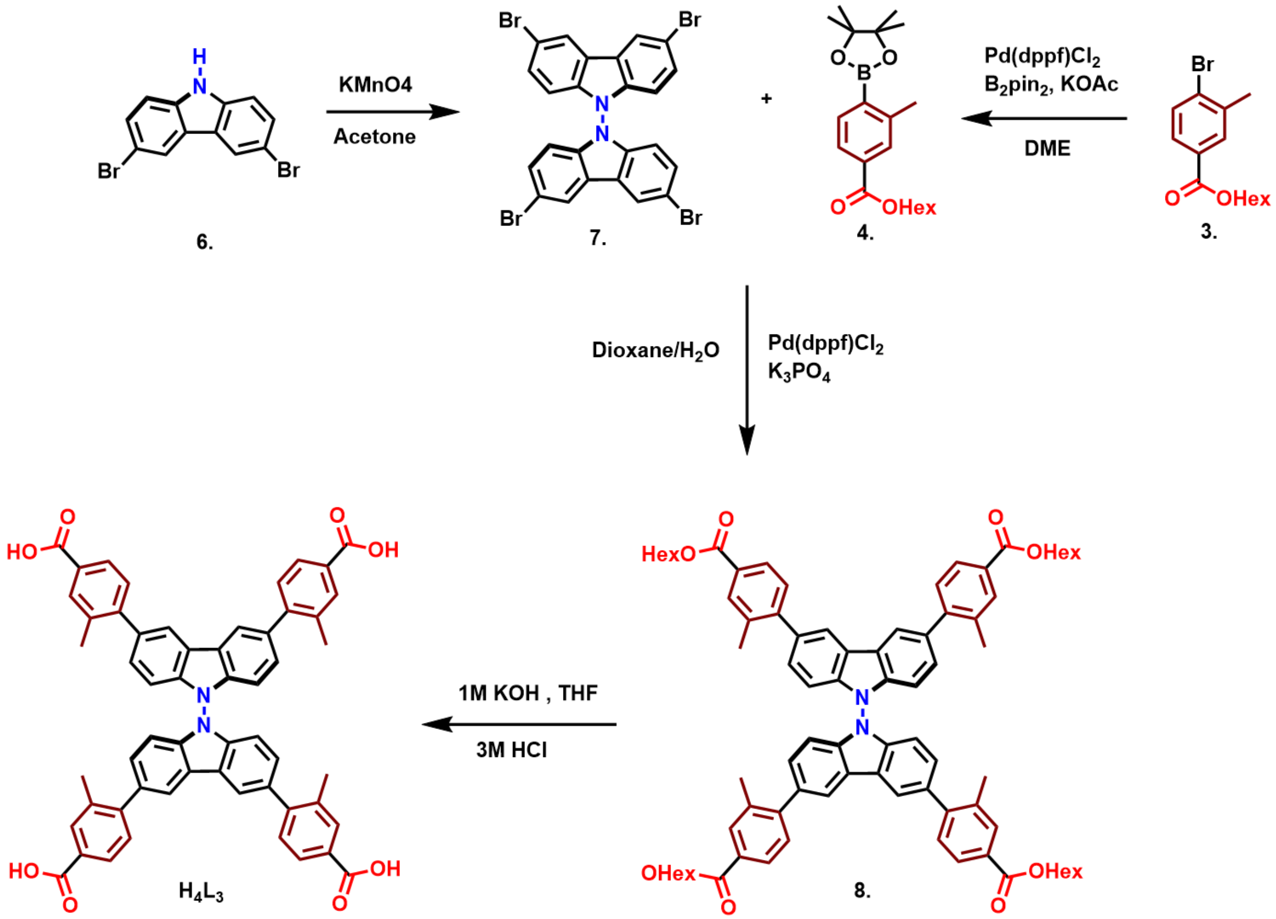

Scheme S2. Synthesis route leading to H_4**L_3**

3,6-dibromo-9H-carbazole (6):

Synthesized according to literature without modification.[3]

3,3',6,6'-tetrabromo-9,9'-bicarbazole (7):

Synthesized according to literature without modification.[4]

4,4',4'',4'''-([9,9'-bicarbazole]-3,3',6,6'-tetrayl)tetrakis(3-methylbenzoic acid) (H_4L_3):

Compound 7 (2.4 g, 3.70 mmol), K_3PO_4 (9.4 g, 44.5 mmol), compound 4 (7.7 g, 22.2 mmol) and Pd(dppf)Cl_2 (0.271 g, 0.37 mmol) were charged in a 250 mL 2-neck round bottom flask equipped with a magnetic stir bar. Dioxane (90 mL) and water (15 mL) was added, and the mixture was degassed by sparring with argon for 30 min. The flask was capped and heated to 90°C under inert atmosphere for 72 h. After cooling down to room temperature, the reaction mixture was extracted into dichloromethane (150 mL × 2) and washed with brine (100 mL). The organic fractions were collected, dried with $MgSO_4$, and concentrated. The off-white solid was

redissolved in DCM, reprecipitated with MeOH and filtered. The solid was transferred to a 500 mL round bottom flask and suspended in 100 mL of THF to which 100 mL of 8 M KOH and 100 mL of MeOH were added. The resulting suspension was stirred under reflux for 48 h. After cooling down to room temperature, the organic solvent was removed in vacuo. The aqueous phase was reprecipitated with 3 M HCl until pH = 2 was reached. The precipitate was filtered, washed with 200 mL of H_2O and dried under vacuum to give H_4L_3 as a colorless solid (1.8 g, yield: 83%).

^{1}H NMR (400 MHz, DMSO) δ 12.90 (s, 4H), 8.50 (s, 5H), 7.90 (d, *J* = 1.3 Hz, 5H), 7.84-7.78 (m, 4H), 7.35 (d, *J* = 7.9 Hz, 5H), 7.30 (d, *J* = 8.5 Hz, 4H), 6.87 (d, J = 8.2 Hz 4H), 2.34 (s, 12H)

^{13}C NMR (150 MHz, DMSO, ppm) δ 167.4, 146.0, 139.0, 135.6, 134.1, 131.3, 130.4 129.5, 127.0, 121.7, 108.6, 20.5

ESI MS found [M+H$^+$] m/z = 869.9526, calc [M+H$^+$] m/z: 869.9434

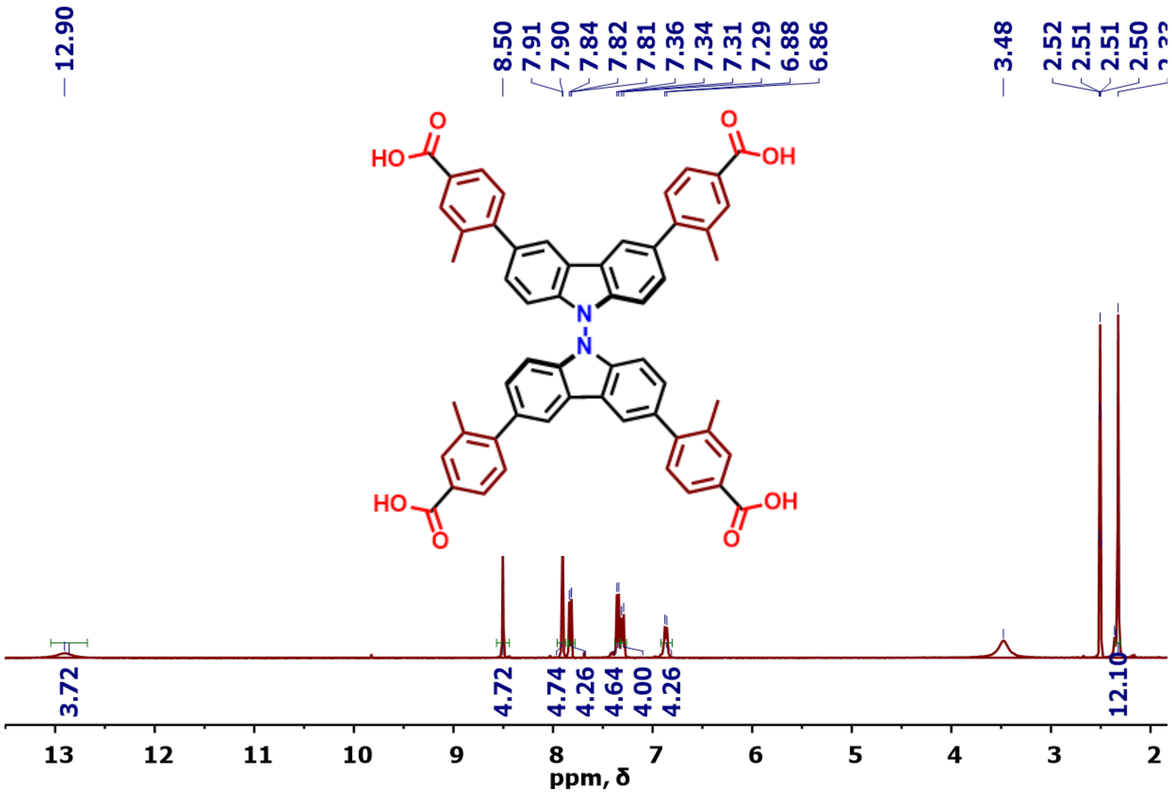

Figure S3. ^{1}H NMR spectrum of $H_4\mathbf{L_3}$

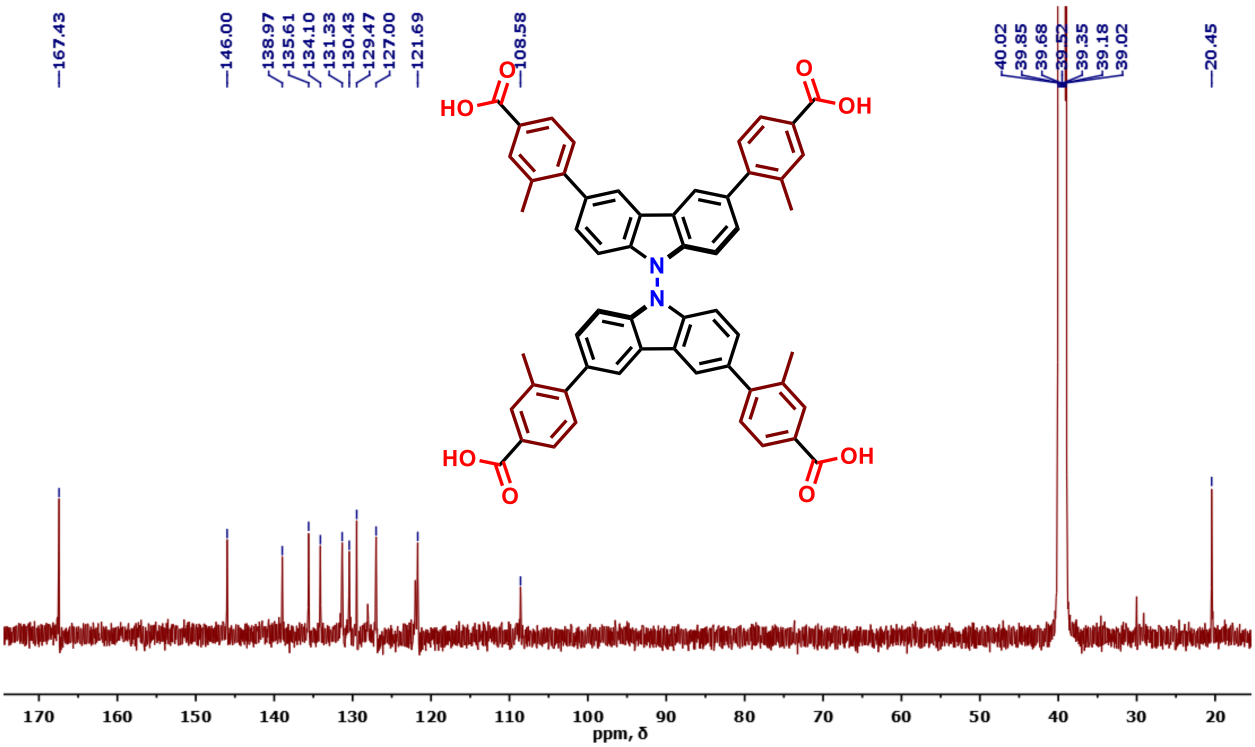

Figure S4. ^{13}C NMR spectrum of $H_4\mathbf{L_3}$

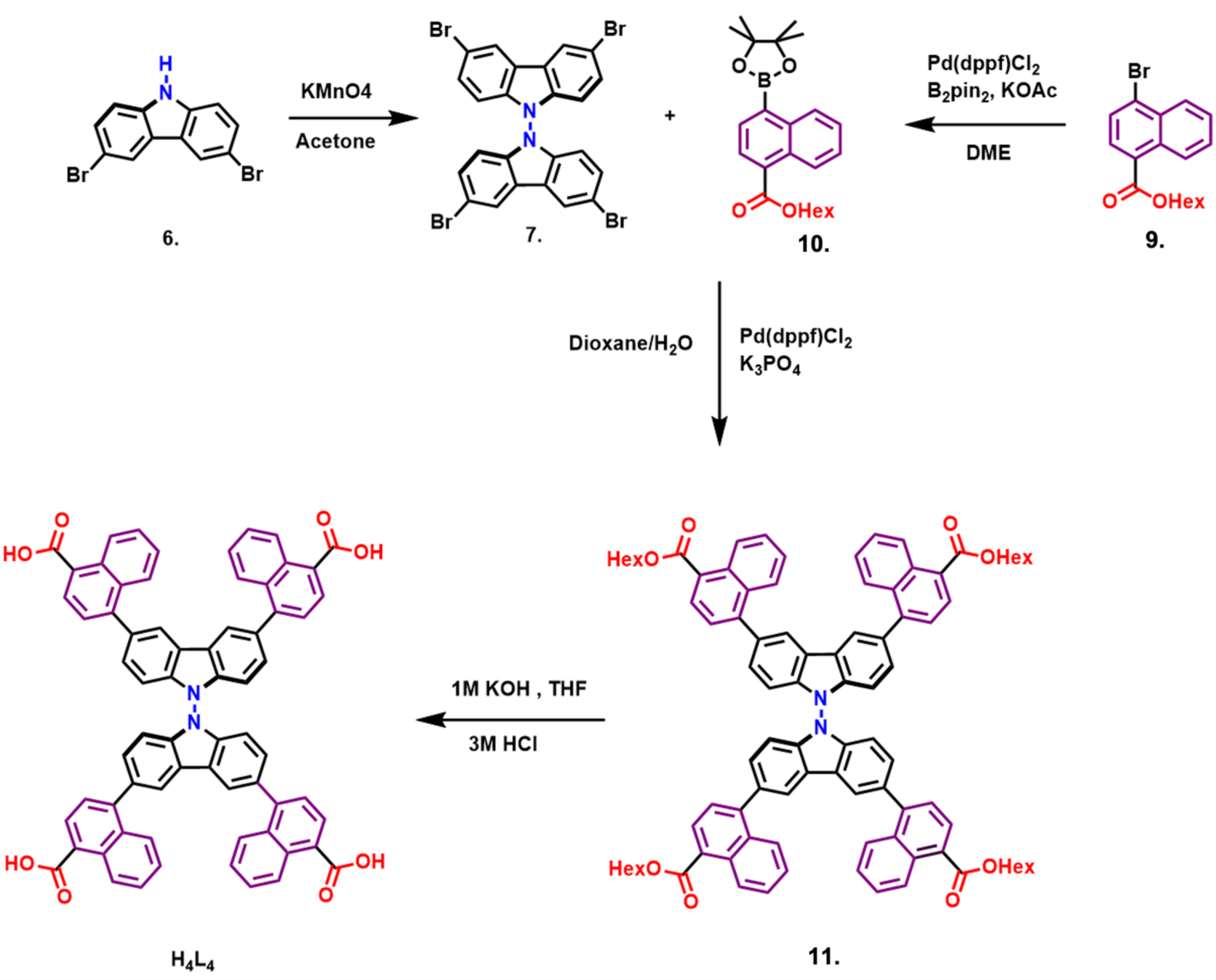

Scheme S3. Synthesis route leading to H_4**L_4**

Hexyl 4-bromo-1-naphthoate (9):

Synthesized according to literature,[5] with the exception that 4-bromo-1-naphthanoic acid was used as the starting material.

^{1}H NMR (400 MHz, $CDCl_3$) δ 9.05-8.73 (m, 1H), 8.40-8.26 (m, 1H), 8.05 (d, *J* = 7.9 Hz, 1H), 7.87 (d, *J* =7.9 Hz, 1H), 7.71-7.60 (m, 2H), 4.26 (t, *J* = 6.7 Hz, 2H), 1.80-1.70 (m, 2H), 1.38 (ddd, *J* = 8.5, 6.1, 3.3 Hz, 9H), 0.97-0.83 (m, 4H).

Hexyl 4-(4,4,5,5-tetramethyl-1,3,2-dioxaborolan-2-yl)-1-naphthoate (10):

Synthesized according to literature,[2] with the exception that hexyl 4-bromo-1-naphthoate was used as the starting material.

^{1}H NMR (400 MHz, $CDCl_3$) δ 9.03-8.89 (m, 1H), 8.41-8.30 (m, 1H), 8.01 (d, *J* = 7.9 Hz, 1H), 7.85 (d, *J* =7.9 Hz, 1H), 7.75-7.60 (m, 2H), 4.43(t, *J* = 6.7 Hz, 2H), 1.92-1.76 (m, 3H), 1.56-1.25 (m, 12H), 1.01-0.86 (m, 5H).

4,4',4'',4'''-([9,9'-bicarbazole]-3,3',6,6'-tetrayl)tetrakis(1-naphthoic acid) (H_4**L_4):**

Compound 7 (2.2 g, 3.40 mmol), K_3PO_4 (7.1 g, 33.6 mmol), compound 4 (7.7 g, 20.4 mmol) and Pd(dppf)Cl_2 (0.248 g, 0.37 mmol) were charged in a 250 mL 2-neck round bottom flask equipped with a magnetic stir bar. Dioxane (90 mL) and water (15 mL) was added, and the mixture was degassed by sparring with argon for 30 min. The flask was capped and heated to 90°C under inert atmosphere for 72 h. After cooling down to room temperature, the reaction mixture was extracted into dichloromethane (150 mL × 2) and washed with brine (100 mL). The organic fractions were collected, dried with $MgSO_4$, and concentrated. The off-white solid was redissolved in DCM, reprecipitated with MeOH and filtered. The solid was transferred to a 500 mL round bottom flask and suspended in 100 mL of THF to which 100 mL of 8 M KOH and 100 mL of MeOH were added. The resulting suspension was stirred under reflux for 48 h. After cooling down to room temperature, the organic solvent was removed in vacuo. The aqueous phase was reprecipitated with 3 M HCl until pH = 2 was reached. The precipitate was filtered, washed with 200 mL of H_2O and dried under vacuum to give H_4L_3 as a colorless solid (1.8 g, yield: 80%).

^{1}H NMR (400 MHz, DMSO) δ 13.32 (s, 4H), 9.01 (d, *J* = 1.3 Hz, 4H), 8.69 (s, 4H). 8.24 (d, J = 7.4 Hz, 4H), 8.03 (d, J = 8.2, 4H) 7.71 – 7.57 (m, 16H), 7.34 (d, J = 8.5 Hz, 4H).

^{13}C NMR (150MHz, DMSO) δ 168.8, 144.6, 139.5, 133.4, 131.9, 131.4, 129.5, 127.5, 127.2, 126.7, 126.7, 126.0, 123.1, 122.0, 109.6

ESI MS found [M+H$^+$] m/z= 1014.0886, calc [M+H$^+$] m/z= 1014.0872

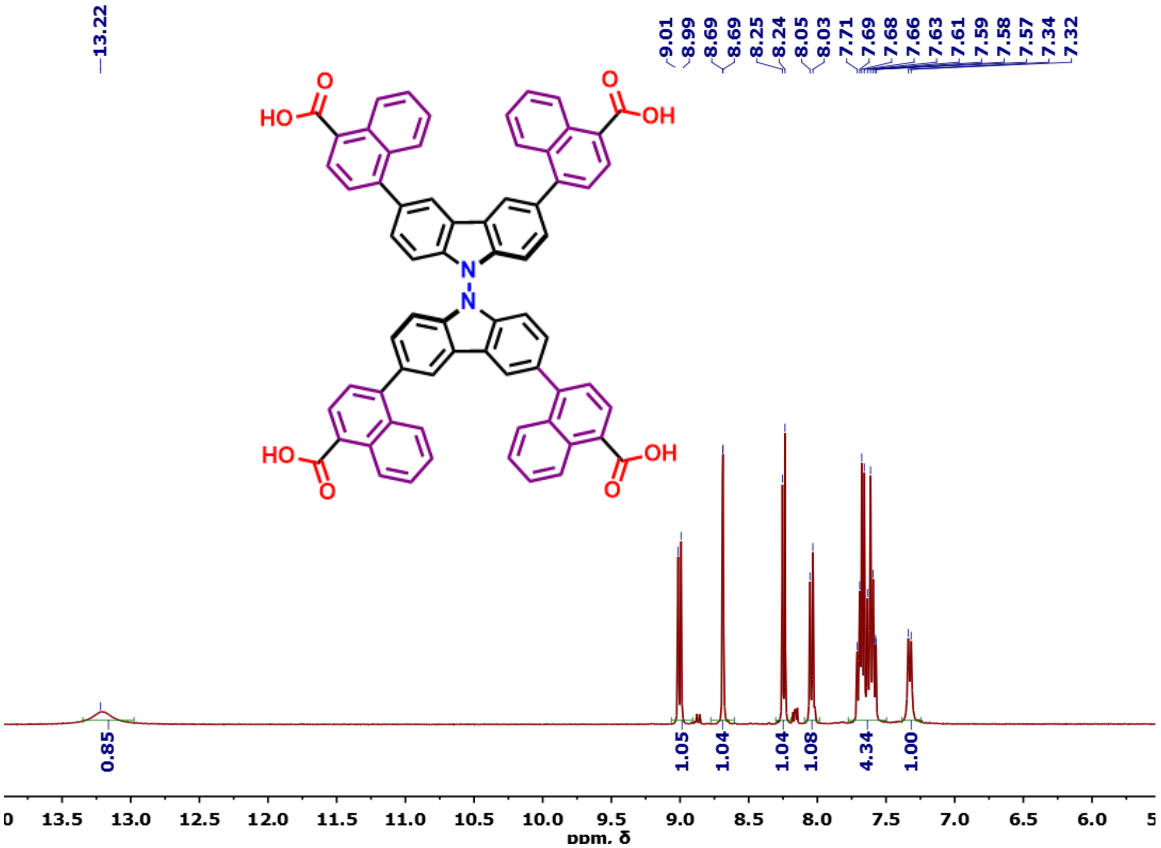

Figure S5. ^{1}H NMR spectrum of H_4**L₄**

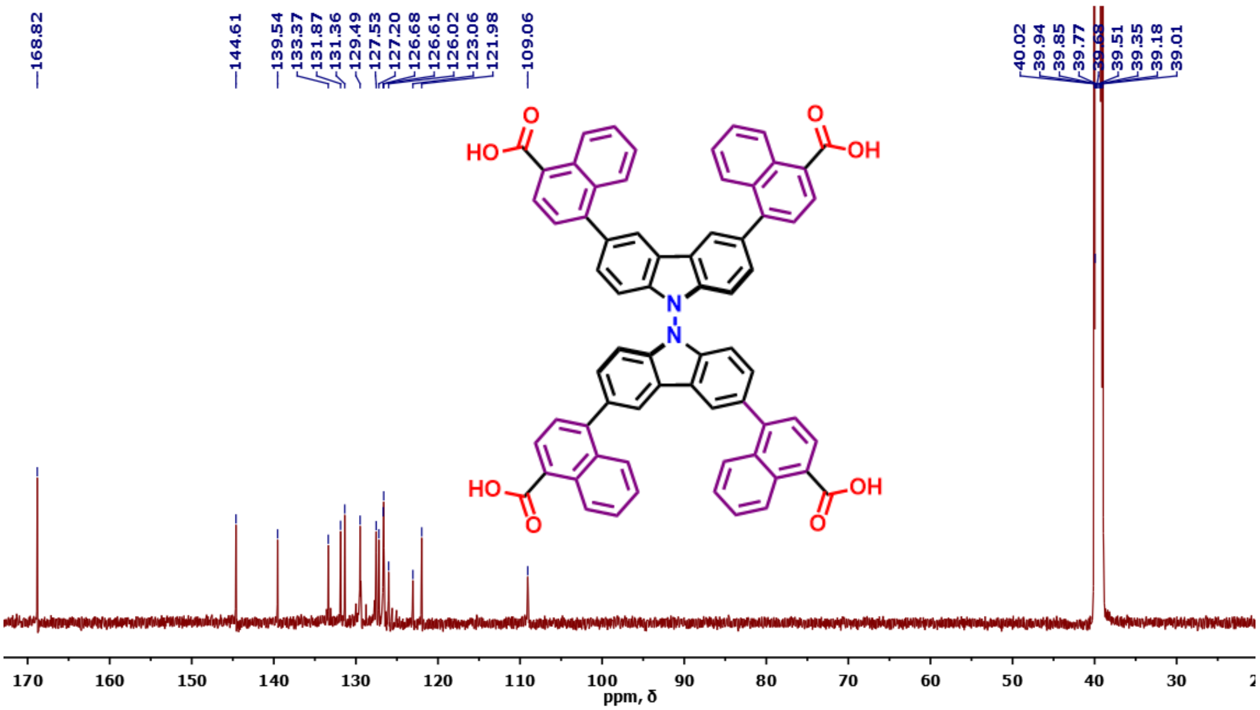

Figure S6. ^{13}C NMR spectrum of H_4**L₄**

Section S-3 MOF Synthesis

NPF-520

$ZrCl_4$ (4.5 mg), and acetic acid (120 µL) were ultrasonically dissolved in 1 mL DMF in a dram vial. The clear solution was heated in an 80°C oven. After 1 h, ligand H_4L_3 (3 mg), added and sonicated for 5 min. The mixture was heated in 120 °C oven for 24 h. After slowly cooling down to room temperature, colorless t hexagonal-shaped crystals were present at the bottom of the vial.

NPF-510

$ZrCl_4$ (4.8 mg), and benzoic acid (90 mg) were ultrasonically dissolved in 1 mL DMF in a dram vial. The clear solution was heated in an 80°C oven. After 1 h, Ligand H_4L_2 (3 mg), and acetic acid (10 µL) were added and sonicated for 5 min. The mixture was heated in a 120°C oven for 24 h. After slowly cooling down to room temperature, colorless truncated octahedron-shaped crystals were present at the bottom of the vial.

NPF-530

$ZrOCl_2$ $8H_2O$ (8.2 mg), and benzoic acid (75 mg) were ultrasonically dissolved in 1 mL DMF in a dram vial. The clear solution was heated in an 80°C oven. After 1 h, Ligand H_4L_4 (3 mg), and trifluoroacetic acid (30 µL) were added and sonicated for 5 min. The mixture was heated in a 120°C oven for 24 h. After slowly cooling down to room temperature, colorless truncated octahedron-shaped crystals were present at the bottom of the vial.

NPF-520-Fe^{III}

In a N_2-filled glovebox, $TMSCH_2Li$ (1.0 M in pentane, 0.2 mL, 20 equiv. w.r.t. Zr_9) was added dropwise to a cold suspension of NPF-520 (0.02 mmol Zr_9) in 20 mL hexanes, and the resultant white suspension was stirred at room temperature for 2 h. The solid was collected through centrifugation and washed with hexanes three times to remove soluble residue. ICP-OES results showed a Li/Zr_9 ratio of 10.9, indicating almost complete lithiation (90%). The resultant NPF-520-Li was then transferred to a vial containing 20 mL of $FeCl_3$ solution (10 mM) in anhydrous CH_3CN. After stirring at room temperature for 2 h, the yellow solid was centrifuged and sonicated with CH_3CN three times. ICP-OES analysis gave a Fe/Zr_9 ratio of 3.2, indicating 3.2 Fe per Zr_9 node.

UiO-66-Fe.

UiO-66 was prepared according to literature procedure. In a N_2-filled glovebox, $TMSCH_2Li$ (1.0 M in pentane, 0.2 mL, 20 equiv. w.r.t. Zr_6) was added dropwise to a cold suspension of UiO-66 (0.01 mmol Zr_6) in 20 mL hexanes, and the resultant white suspension was stirred at room temperature for 2 h. The solid was collected through centrifugation and washed with hexanes three times to remove soluble residue. The resultant UiO-66-Li was then transferred to a vial containing 20 mL of $FeCl_3$ solution (10 mM) in anhydrous CH_3CN. After stirring at room temperature for 2 h, the yellow solid was centrifuged and sonicated with CH_3CN three times. ICP-OES analysis gave a Fe/Zr_6 ratio of 1.2, indicating 1.2 Fe per Zr_6 node.

UiO-69-Fe.

UiO-69 was prepared according to literature procedure. In a N_2-filled glovebox, $TMSCH_2Li$ (1.0 M in pentane, 0.2 mL, 20 equiv. w.r.t. Zr_6) was added dropwise to a cold suspension of UiO-69 (0.01 mmol Zr_6) in 20 mL hexanes, and the resultant white suspension was stirred at room temperature for 2 h. The solid was collected through centrifugation and washed with hexanes three times to remove soluble residue. The resultant UiO-69-Li was then transferred to a vial containing 20 mL of $FeCl_3$ solution (10 mM) in anhydrous CH_3CN. After stirring at room temperature for 2 h, the yellow solid was centrifuged and sonicated with CH_3CN three times. ICP-OES analysis gave a Fe/Zr_6 ratio of 1.2, indicating 1.2 Fe per Zr_6 node.

Section S-4 Optical Microscopic Images

a.

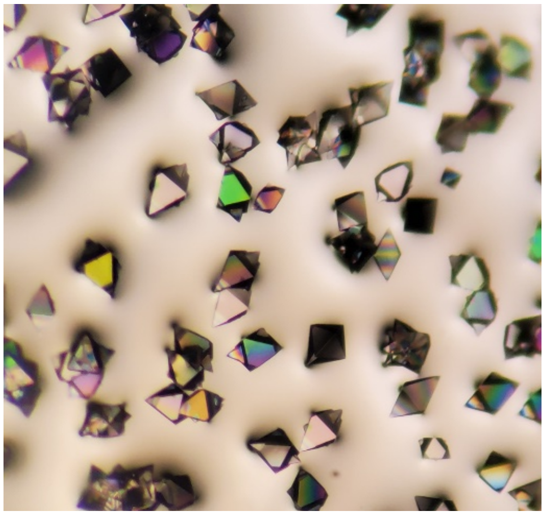

b.

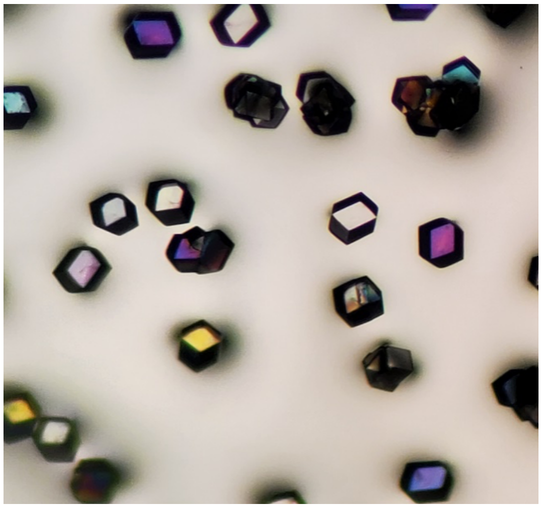

c.

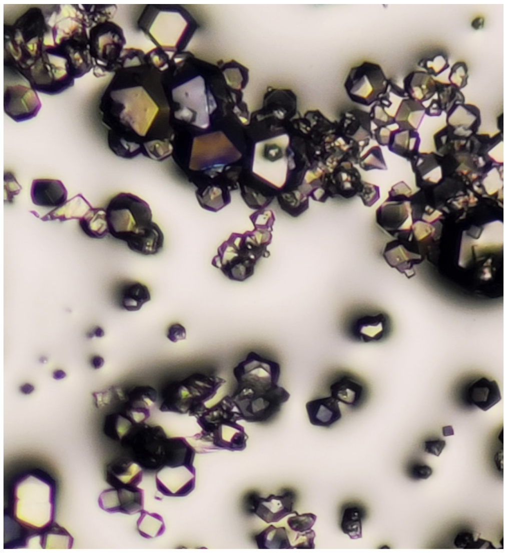

Figure S7. Single crystals of as-synthesized a) **NPF-510**, b) **NPF-520**, c) **NPF-530.**

Section S-5 Crystallographic Data and Structural Representation

Single crystal X-ray diffraction data were collected using synchrotron radiation at the Advanced Light Source, Berkeley CA. Indexing was performed using APEX3 (Difference Vectors method).[6] Data integration and reduction were performed using SaintPlus 6.0.[7] Absorption correction was performed by multi-scan method implemented in SADABS.[8] Space groups were determined using XPREP implemented in APEX3.[9] The structure was solved using SHELXS-97 (direct methods) and refined using SHELXL-97 within Olex 2 (full-matrix least-squares on F^2).[10, 11] Zr, C, O atoms were refined with anisotropic displacement parameters and H atoms were placed in geometrically calculated positions and included in the refinement process using riding model with isotropic thermal parameters: $U_{iso}(H) = 1.2U_{eq}(-CH)$. The disordered solvent molecules were treated as diffuse using the SQUEEZE procedure implemented in PLATON.[12] Crystal data and refinement details are shown in Table S1, S2, S3 S4 and this data can be obtained free of charge from The Cambridge Crystallographic Data Centre via www.ccdc.cam.ac.uk/data_request/cif

Table S1. Crystal data and structure refinement for NPF-510

Empirical formula	$C_{114}H_{80}O_{32}Zr_6$
Formula weight	2509.10
Temperature/K	273(2)
Crystal system	tetragonal
Space group	*I*4/*mmm*
a/Å	20.1900(9)
b/Å	20.1900(9)
c/Å	41.707(2)
α/°	90
β/°	90
γ/°	90
Volume/Å^3	17001.1(18)
Z	2
ρ_{calc}/g cm^{-3}	0.490
μ/mm^{-1}	0.217
F(000)	2520.0
Crystal size/mm^3	0.4 × 0.3 × 0.25
Radiation	synchrotron (λ = 0.7288)
2Θ range for data collection/°	2.298 to 49.518
Index ranges	-23 ⩽ h ⩽ 23, -23 ⩽ k ⩽ 23, -47 ⩽ l ⩽ 47
Reflections collected	134401
Independent reflections	3816 [R_{int} = 0.0971, R_{sigma} = 0.0656]
Data/restraints/parameters	3816/22/139
Goodness-of-fit on F^2	1.074
Final *R* indexes [I>=2σ (I)]	R_1 = 0.1072, wR_2 = 0.2755
Final *R* indexes [all data]	R_1 = 0.1612, wR_2 = 0.3288
Largest diff. peak/hole / e Å^{-3}	0.86/-0.81

Table S2. Crystal data and structure refinement for NPF-520

Empirical formula	$C_{171}H_{129}N_6O_{45}Zr_9$
Formula weight	3808.77
Temperature/K	273(2)
Crystal system	trigonal
Space group	*R*32
a/Å	35.168(9)
b/Å	35.168(9)
c/Å	28.593(12)
α/°	90
β/°	90
γ/°	120
Volume/Å^3	30625(21)
Z	3
ρ_{calc}/g cm^{-3}	0.620
μ/mm^{-1}	0.271
F(000)	5751.0
Crystal size/mm^3	0.4 × 0.4 × 0.28
Radiation	synchrotron (λ = 0.7288)
2Θ range for data collection/°	2.004 to 57.292
Index ranges	-46 ≤ h ≤ 44, -45 ≤ k ≤ 46, -34 ≤ l ≤ 37
Reflections collected	100902
Independent reflections	16078 [R_{int} = 0.1060, R_{sigma} = 0.0748]
Data/restraints/parameters	16078/293/340
Goodness-of-fit on F^2	1.079
Final *R* indexes [I>=2σ (I)]	R_1 = 0.1172, wR_2 = 0.2981
Final *R* indexes [all data]	R_1 = 0.1857, wR_2 = 0.3863
Largest diff. peak/hole / e Å^{-3}	4.94/-2.42

Table S3. Crystal data and structure refinement for NPF-530.

Empirical formula	$C_{136}H_{72}N_4O_{32}Zr_6$
Formula weight	2821.29
Temperature/K	273(2)
Crystal system	tetragonal
Space group	*I*4/*mmm*
a/Å	19.5892(9)
b/Å	19.5892(9)
c/Å	44.128(3)
α/°	90
β/°	90
γ/°	90
Volume/Å^3	16933.5(19)
Z	2
ρ_{calc}/g cm^{-3}	0.553
μ/mm^{-1}	0.222
F(000)	2824.0
Crystal size/mm^3	0.2 × 0.3 × 0.35
Radiation	synchrotron (λ = 0.7288)
2Θ range for data collection/°	3.55 to 41.42
Index ranges	-19 ≤ h ≤ 19, -18 ≤ k ≤ 18, -42 ≤ l ≤ 42
Reflections collected	50576
Independent reflections	2289 [R_{int} = 0.1080, R_{sigma} = 0.0375]
Data/restraints/parameters	2289/214/153
Goodness-of-fit on F^2	1.064
Final *R* indexes [I>=2σ (I)]	R_1 = 0.1991, wR_2 = 0.3670
Final *R* indexes [all data]	R_1 = 0.2599, wR_2 = 0.3970
Largest diff. peak/hole / e Å^{-3}	1.88/-1.28

Table S4. Crystal data and structure refinement for NPF-520-Fe^{III}.

Empirical formula	$C_{168}H_{120}N_6O_{45}Fe_{2.88}Zr_9$
Formula weight	3924.52
Temperature/K	273(2)
Crystal system	trigonal
Space group	*R*32
a/Å	34.311(6)
b/Å	34.311(6)
c/Å	28.783(8)
α/°	90
β/°	90
γ/°	120
Volume/$Å^3$	29345(13)
Z	3
ρ_{calc}/g cm^{-3}	0.666
μ/mm^{-1}	0.392
F(000)	5895.0
Crystal size/mm^3	0.3 × 0.3 × 0.18
Radiation	synchrotron (λ = 0.7288)
2Θ range for data collection/°	2.434 to 50.768
Index ranges	-40 ⩽ h ⩽ 40, -40 ⩽ k ⩽ 40, -33 ⩽ l ⩽ 33
Reflections collected	137436
Independent reflections	11149 [R_{int} = 0.1224, R_{sigma} = 0.1275]
Data/restraints/parameters	11149/194/352
Goodness-of-fit on F^2	0.973
Final *R* indexes [I>=2σ (I)]	R_1 = 0.0953, wR_2 = 0.2368
Final *R* indexes [all data]	R_1 = 0.1580, wR_2 = 0.3101
Largest diff. peak/hole / e $Å^{-3}$	1.89/-1.19

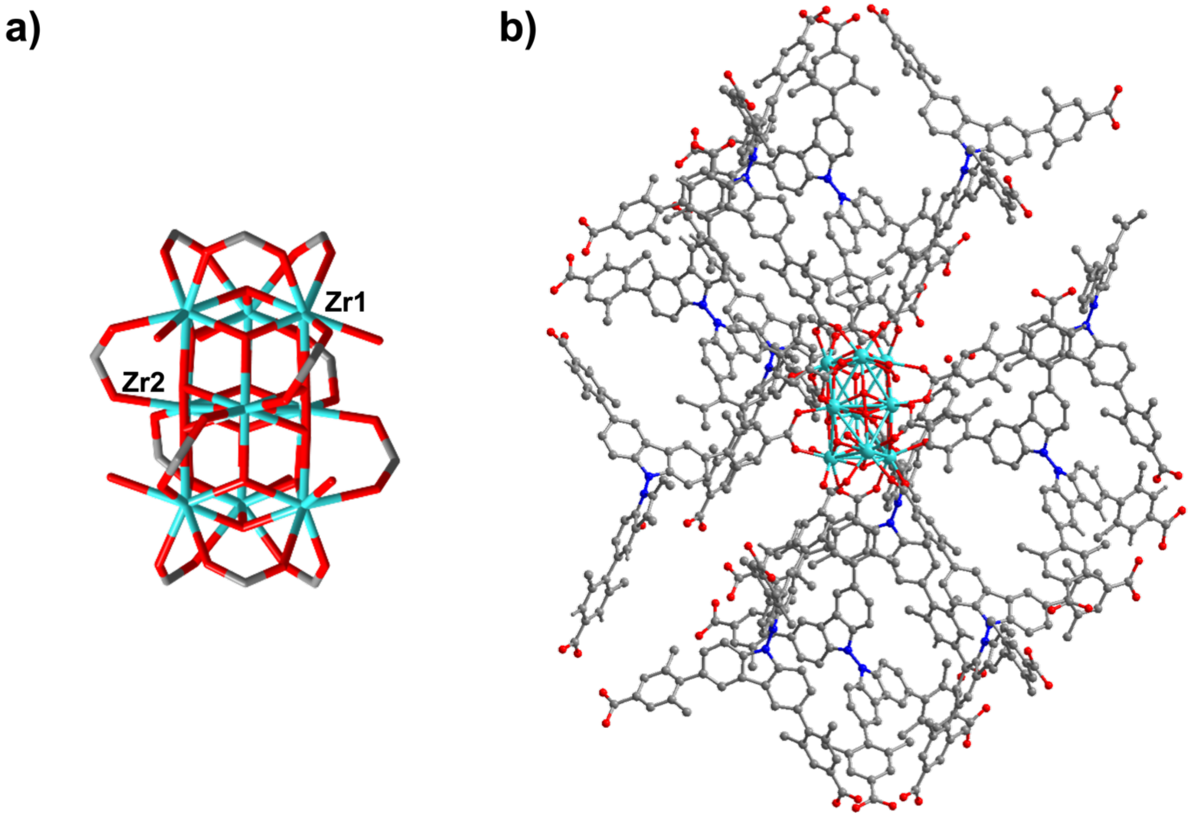

Figure S8. (a) Structure and (b) connectivity of the Zr_9 cluster in NPF-520.

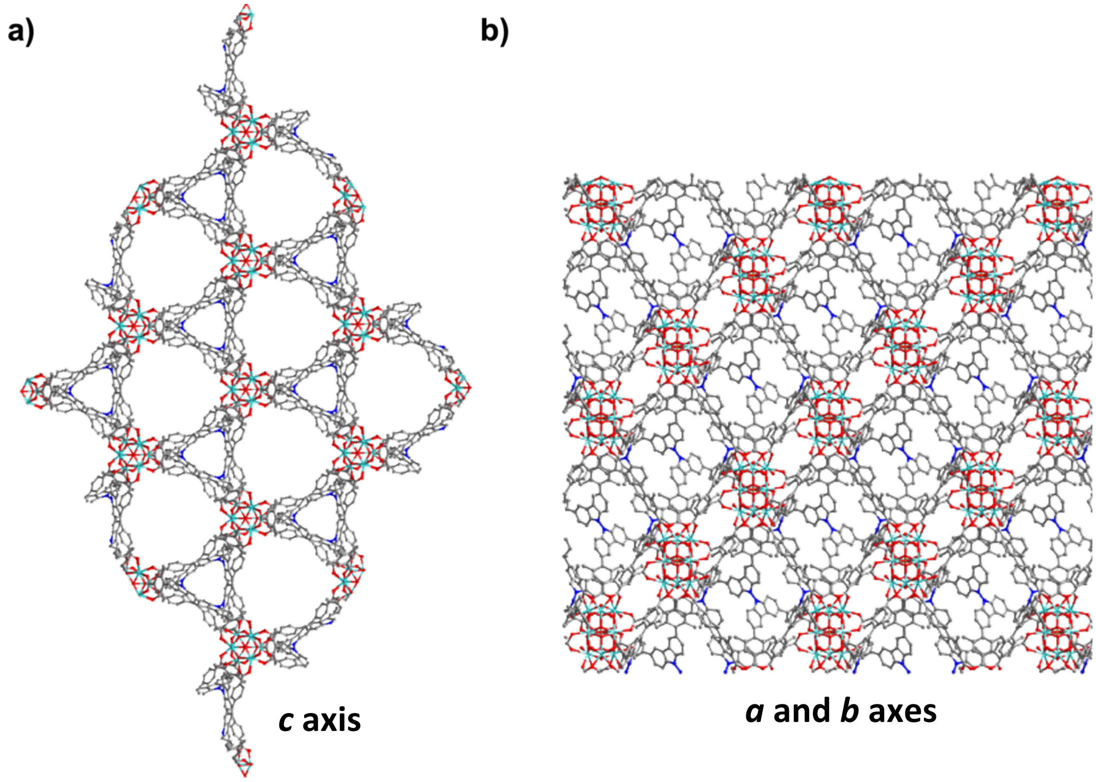

Figure S9. Packing scheme of NPF-520 along *c* and *a/b* axes.

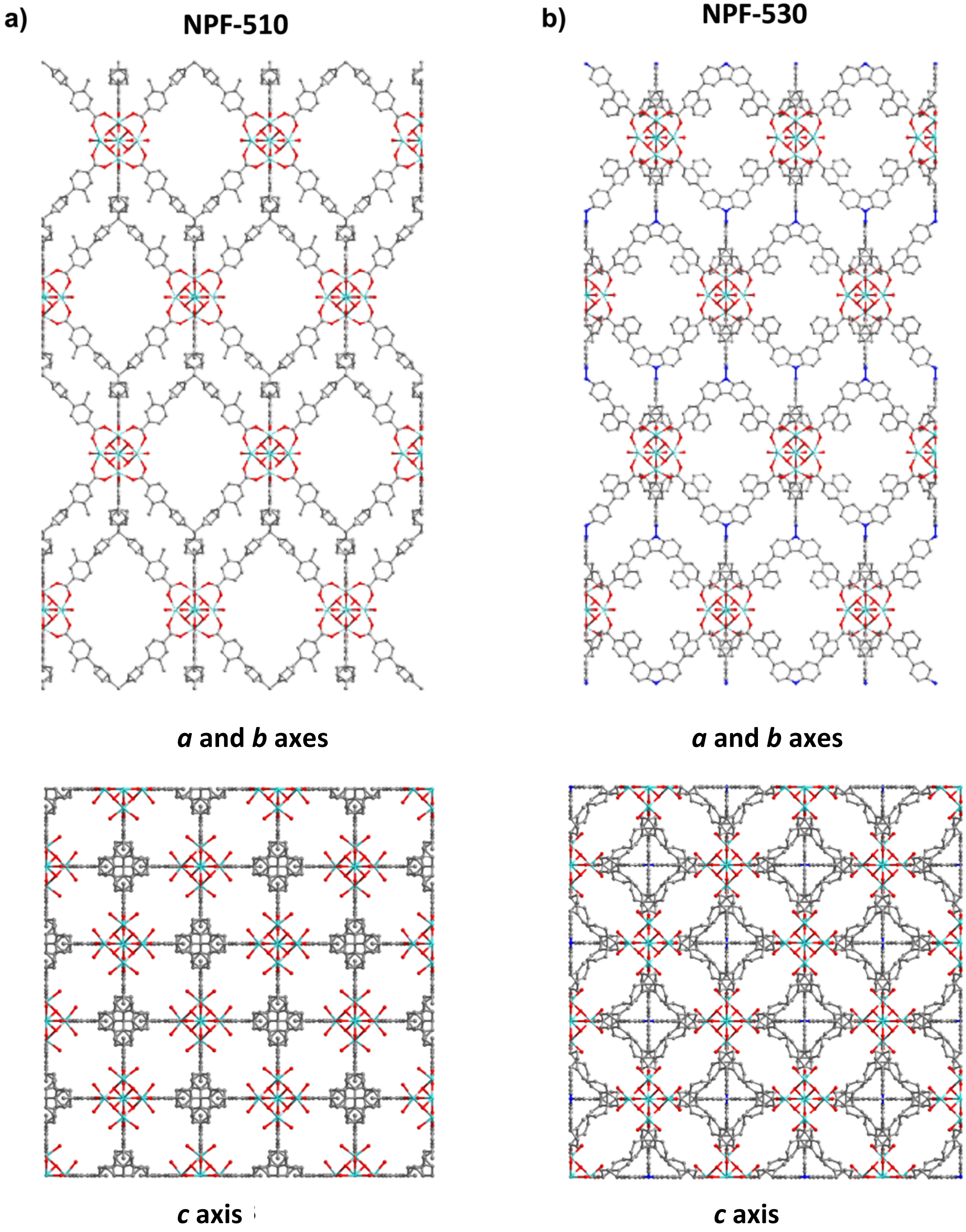

Figure S10. Packing scheme of (a) NPF-510 and (b) NPF-530 along *c* and *a/b* axes.

Section S-6 Thermogravimetric Analysis

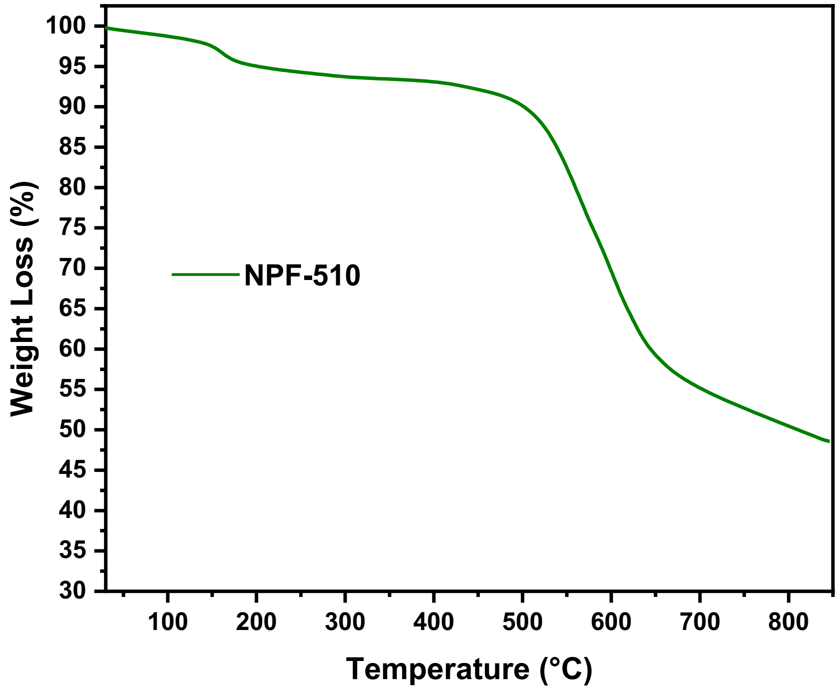

Figure S11. TGA curve of desolvated NPF-510

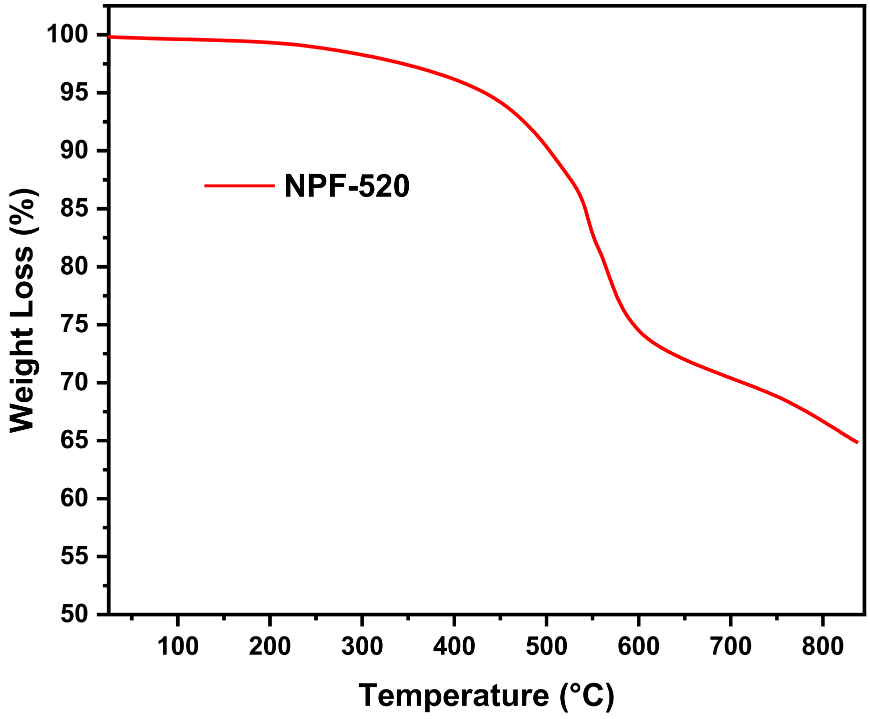

Figure S12. TGA curve of desolvated NPF-520

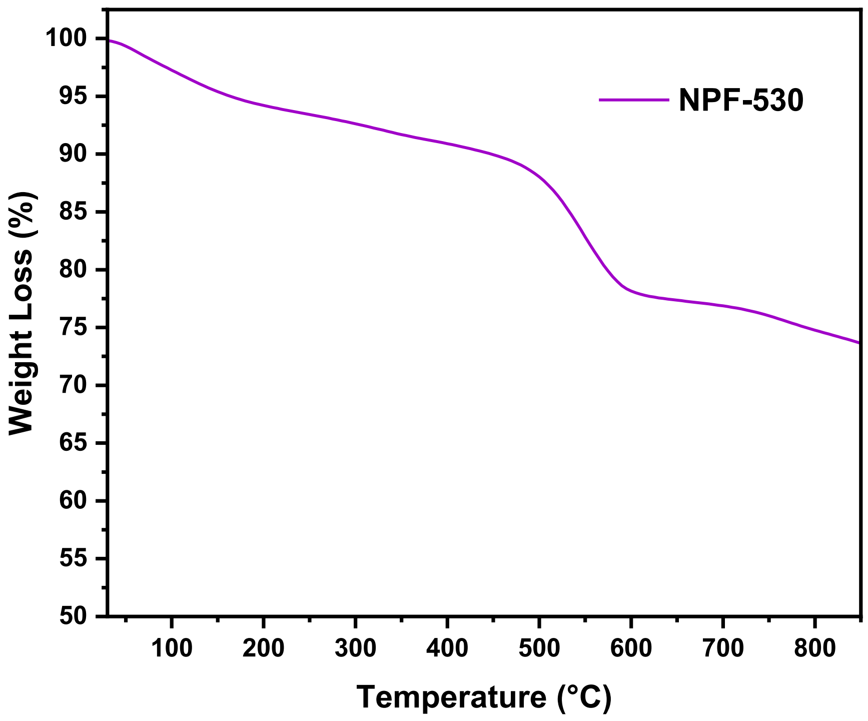

Figure S13. TGA curve of desolvated NPF-530

Section S-7 Powder X-ray Diffraction

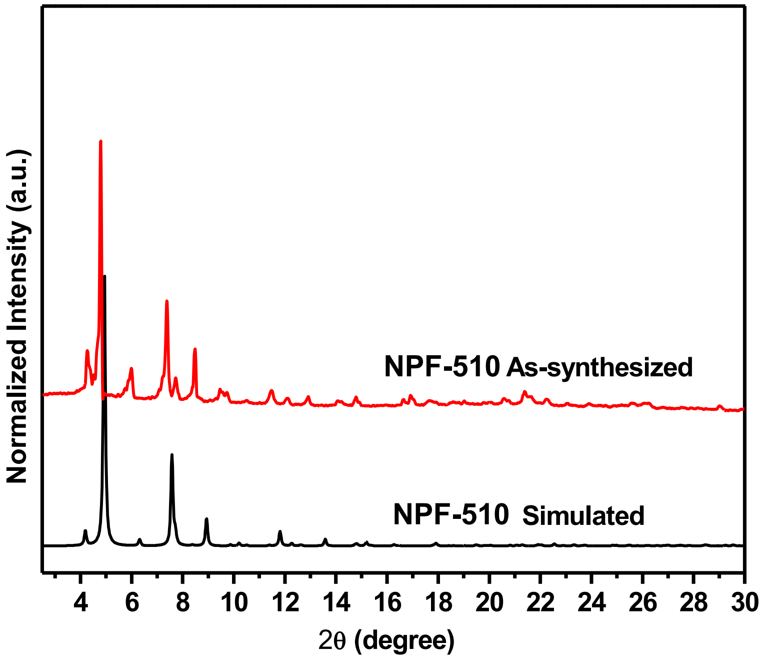

Figure S14. PXRD patterns of NPF-510

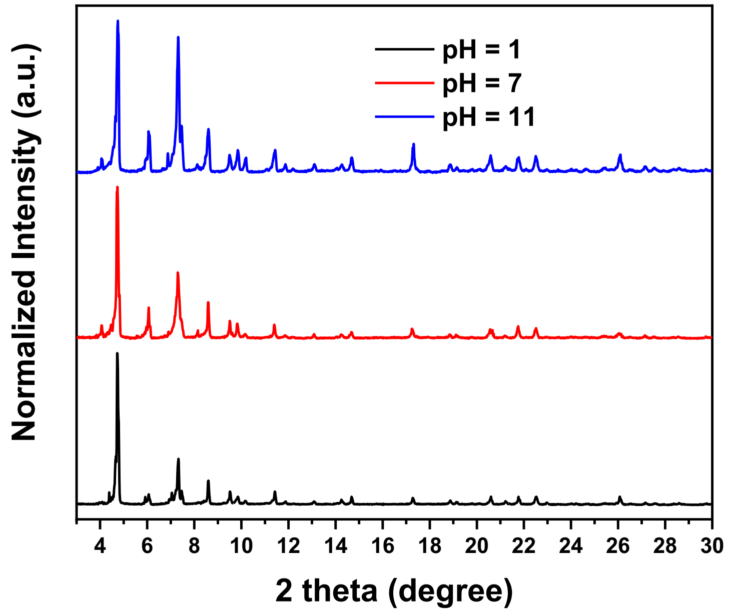

Figure S15. PXRD patterns of NPF-510 after acid, water, and base treatment

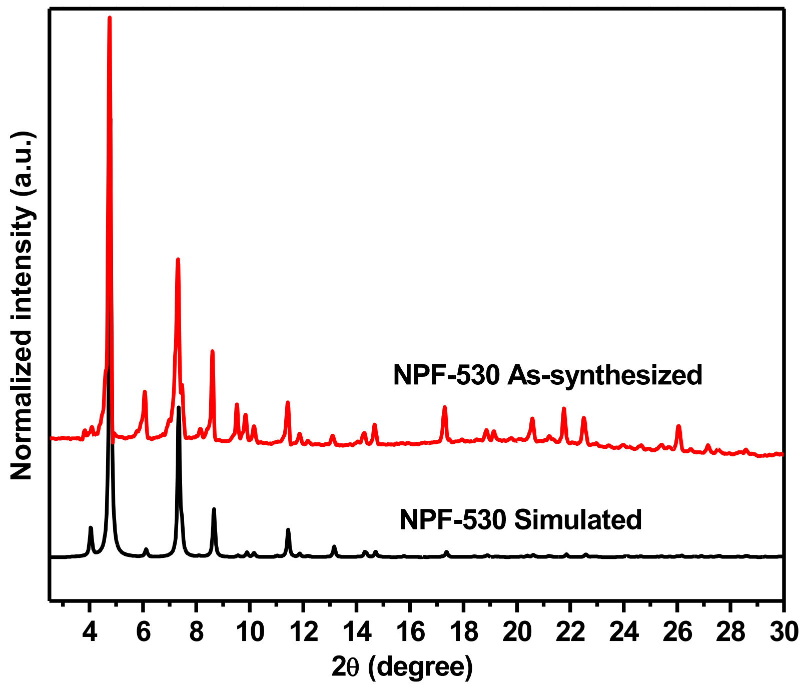

Figure S16. PXRD patterns of NPF-530

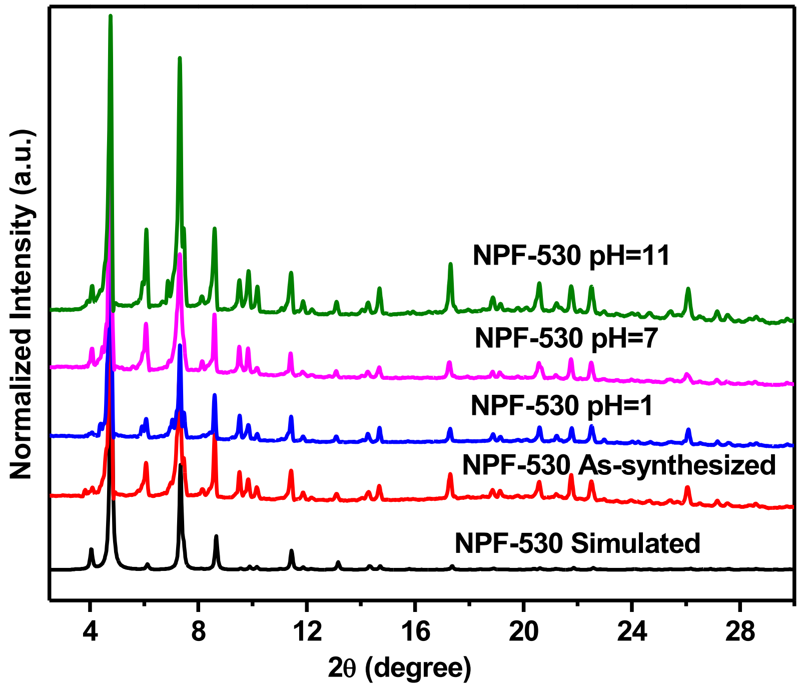

Figure S17. PXRD patterns of NPF-530 after acid, water, and base treatment

Section S-8 N_2 Sorption Isotherms

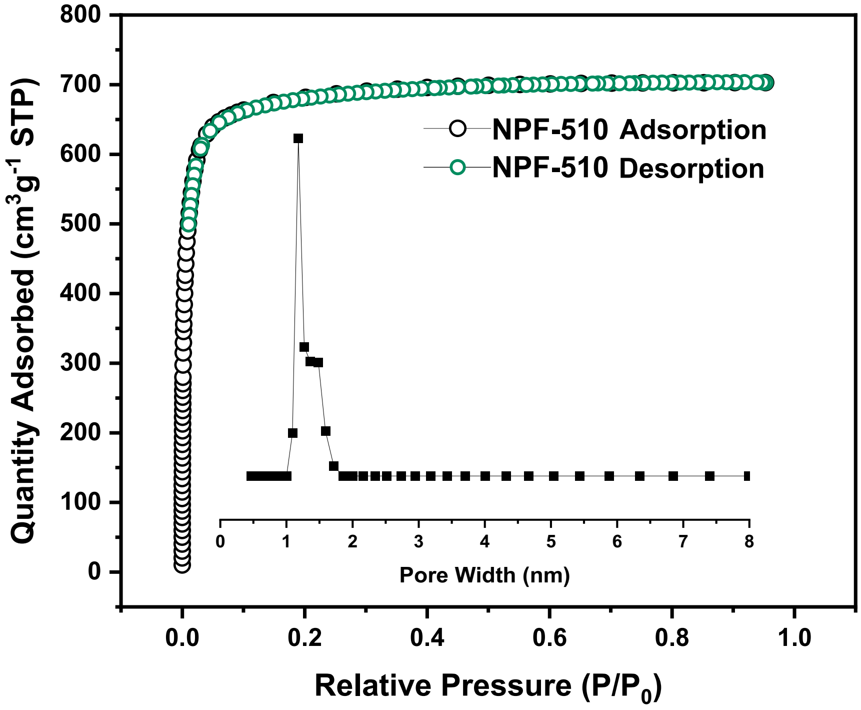

Figure S18. N_2 sorption isotherm at 77 K and DFT pore size distribution of **NPF-510** (BET Surface Area: 2674 m^2g^{-1})

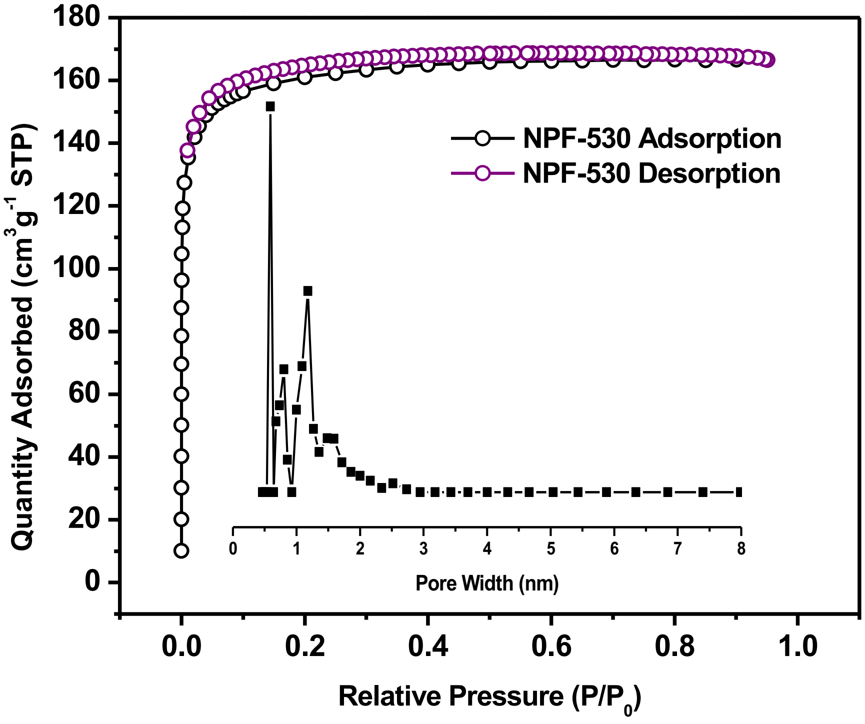

Figure S19. N_2 sorption isotherm at 77 K and DFT pore size distribution of **NPF-530** (BET Surface Area: 619 m^2g^{-1})

Section S-9 Characterization of NPF-520-Fe^{III}

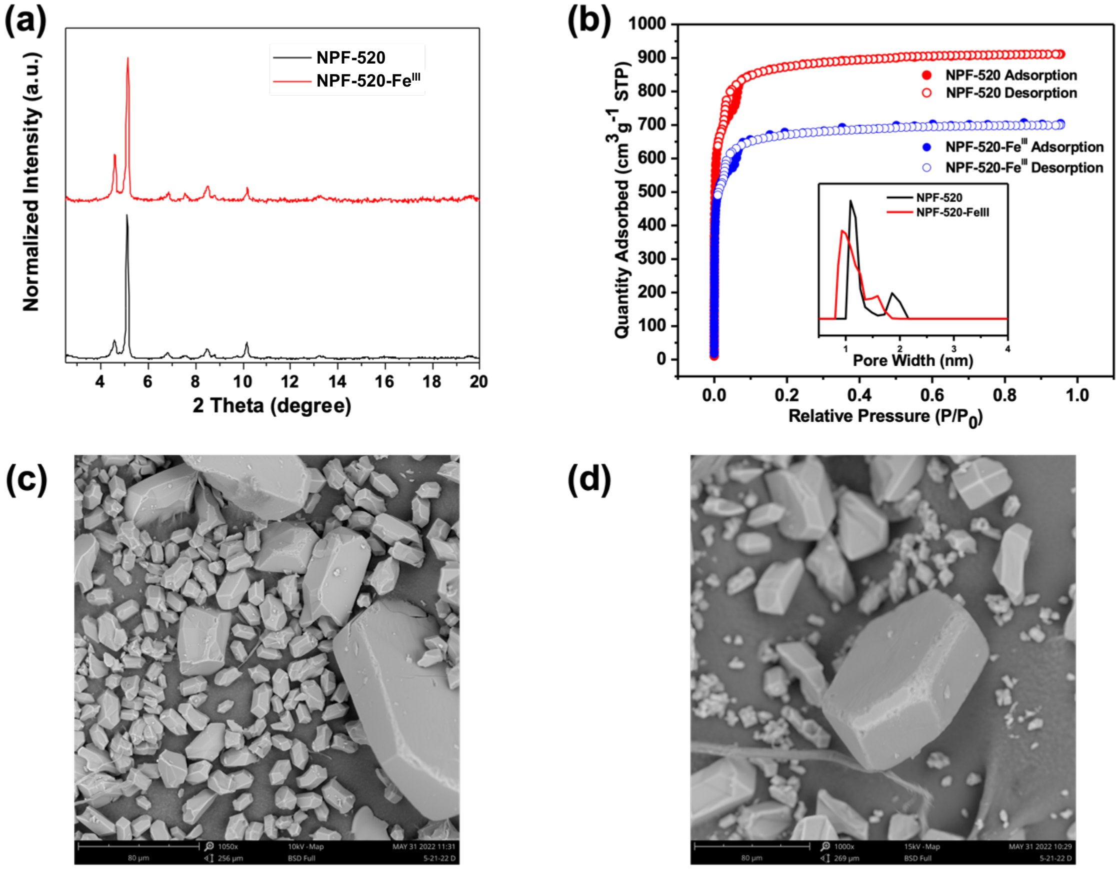

Figure S20. (a) Powder X-ray diffraction patterns and (b) adsorption/desorption isotherms and pore size distribution, (c-d) SEM images of NPF-520 and NPF-520-Fe^{III}.

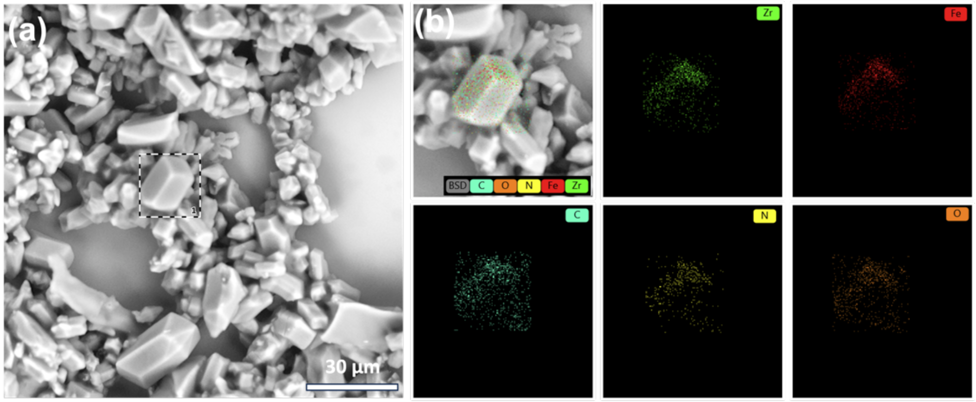

Figure S21. (a) SEM and (b) energy dispersive X-ray (EDX) mapping analysis of NPF-520-Fe^{III}.

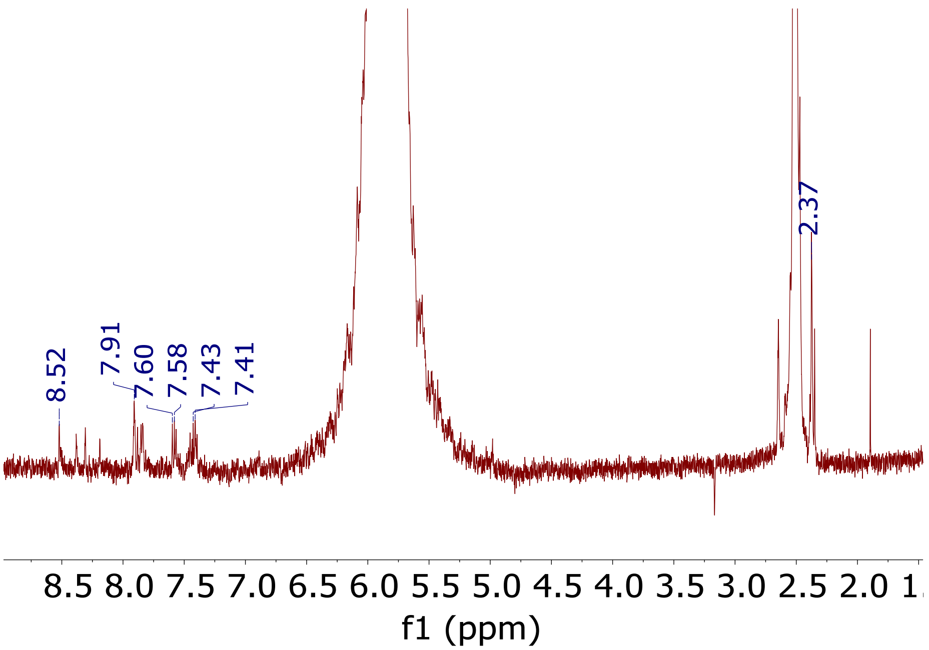

Figure S22. ^{1}H NMR of as-synthesized NPF-520-Fe^{III}, digested in D_2SO_4/d_6-DMSO.

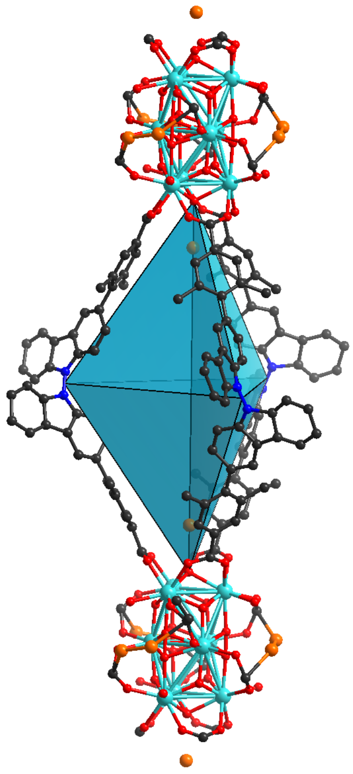

Figure S23. Trigonal bipyramidal cage in NPF-520-Fe^{III}.

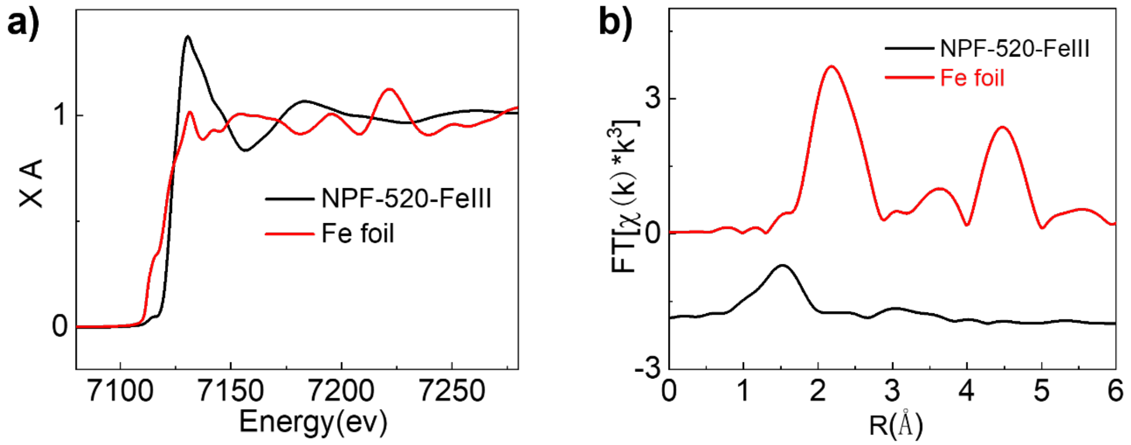

Figure S24. X-ray absorption near edge (XANES) spectrum of Fe foil reference and NPF-520-Fe^{III} and their Fourier transformed XAFS.

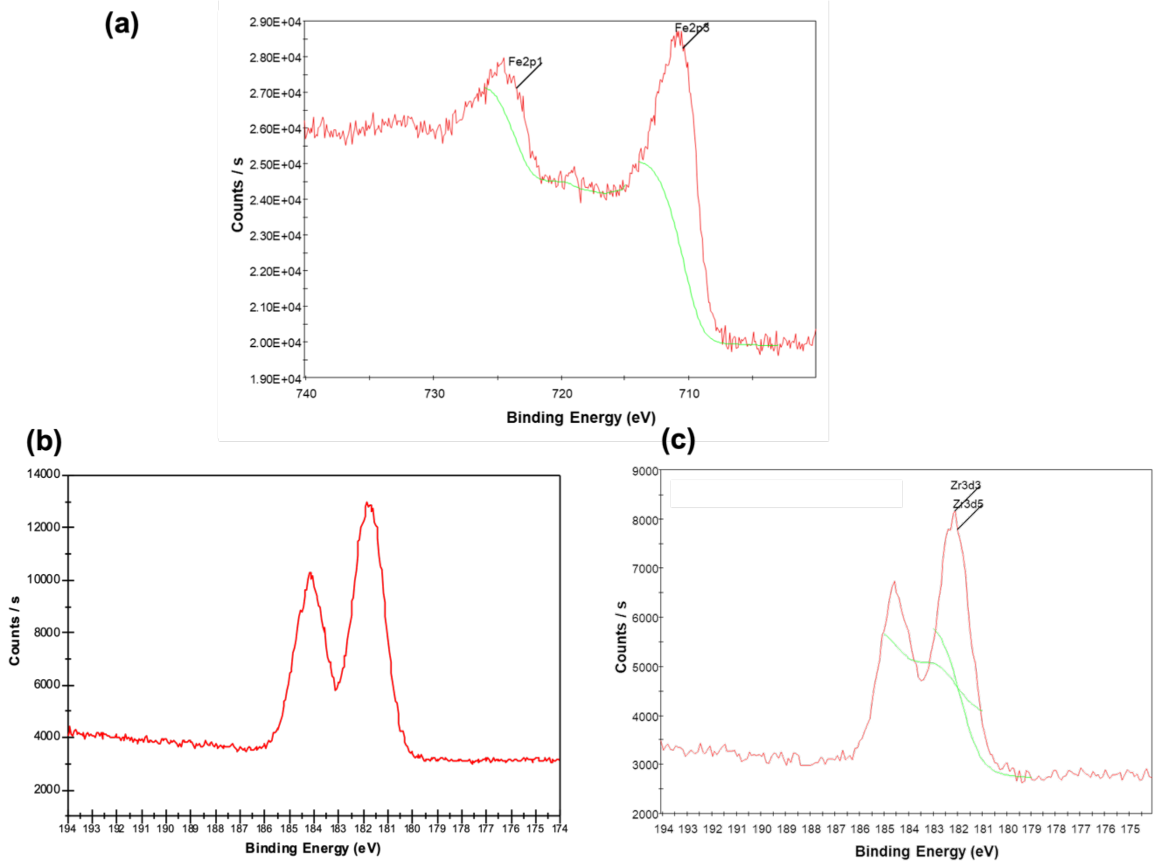

Figure S25. (a) Fe 2p and (b-c) Zr 3d XPS of NPF-520 and NPF-520-Fe^{III}.

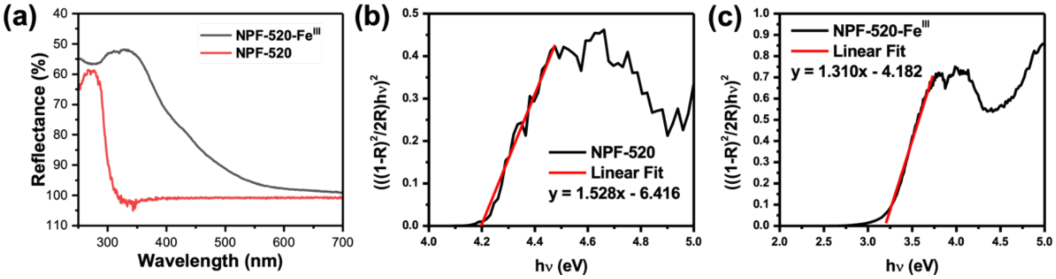

Figure S26. (a) UV-vis reflectance spectra of NPF-520 and NPF-520-Fe^{III}. (b) Tauc plot for NPF-520. (c) Tauc plot for NPF-520-Fe^{III}.

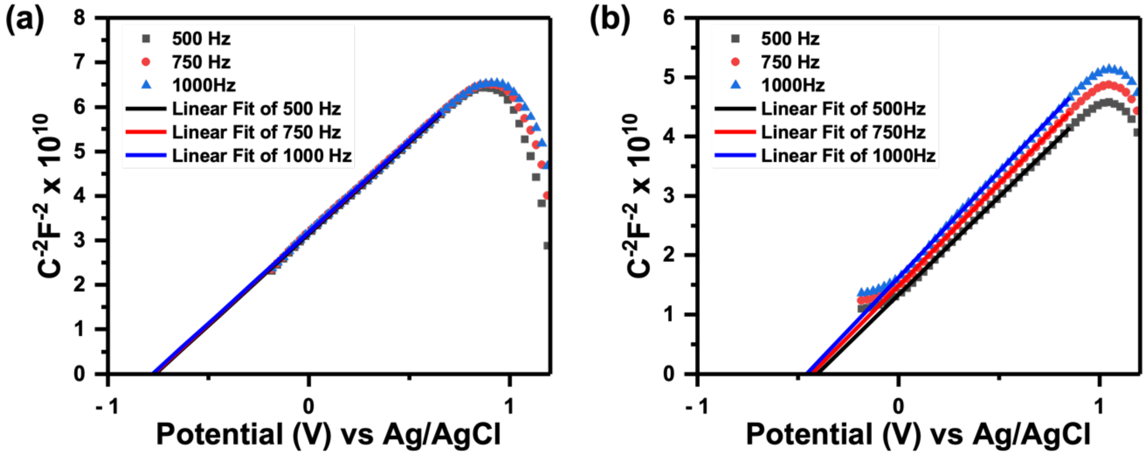

Figure S27. Mott-Schottky plots for (a) NPF-520 and (b) NPF-520-Fe^{III}.

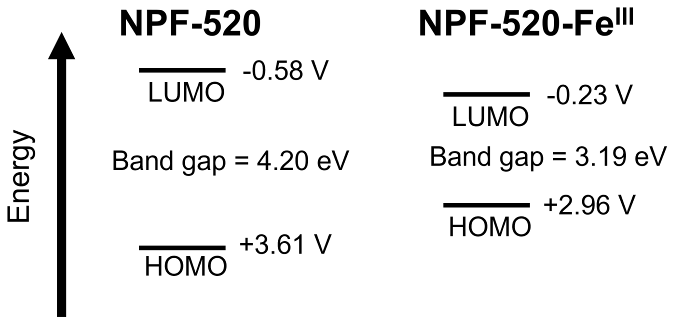

Figure S28. Energy level diagram for NPF-520 and NPF-520-Fe^{III}.

Section S-10 Photocatalysis

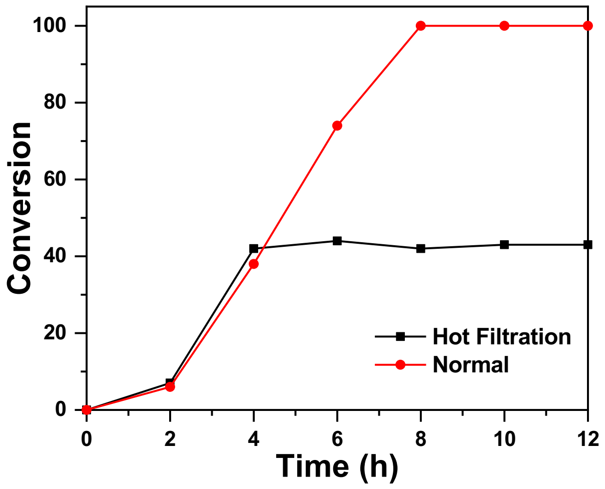

Figure S29. Hot filtration of NPF-520-Fe^{III}.

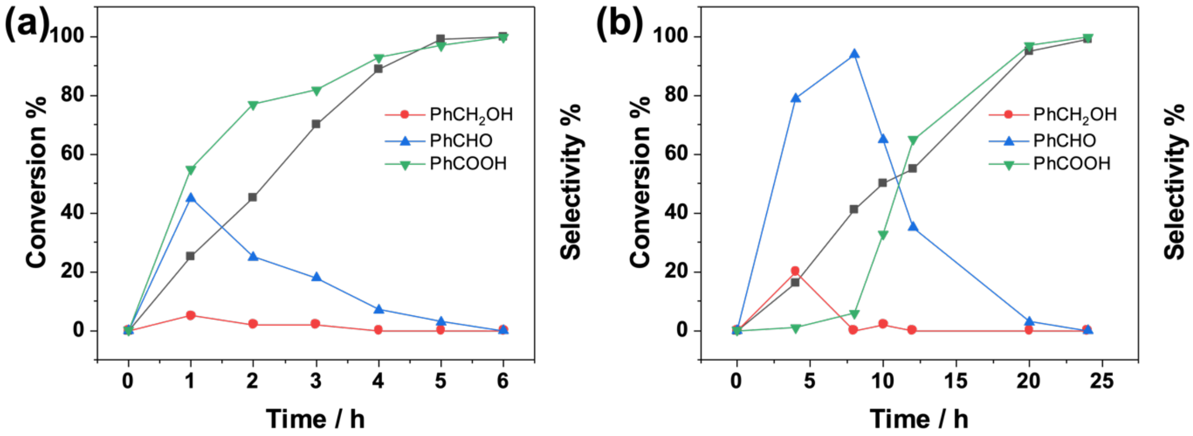

Figure S30. Time dependent product monitoring for (a) 20 µL and (b) 100 µL water.

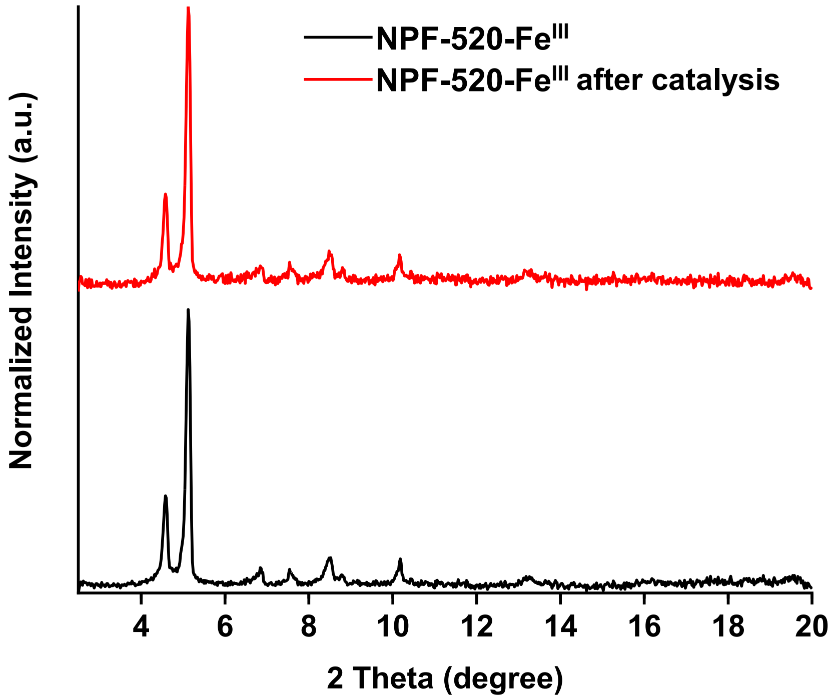

Figure S31. PXRD patterns of NPF-520-Fe^{III} before and after catalysis (anhydrous).

Table S5. Catalyst Recycling and Leaching

Entry	Catalyst	Times Recycled	Conversion[c]	Fe/Zr_9 Ratio (ICP-OES)
1[a]	NPF-520-Fe^{III}	0	100	3.14
2[a]	NPF-520-Fe^{III}	1	100	3.10
3[a]	NPF-520-Fe^{III}	2	98	3.10
4[a]	NPF-520-Fe^{III}	3	99	3.09
5[a]	NPF-520-Fe^{III}	4	100	3.11
6[a]	NPF-520-Fe^{III}	5	97	3.09
7[ad]	NPF-520-Fe^{III}	6	92	2.98

[a]Reaction conditions: 5 µL toluene, 1 mL MeCN, 5 mol% catalyst (based on Fe^{III}), 395 nm blue LED photoreactor, 8 h, 1 atm O_2. [b]Reaction conditions: 10 µL toluene, 1 mL MeCN, 1 mol% catalyst (based on Fe^{III}), 395 nm blue LED photoreactor, 2 h, 1 atm O_2. [c]Determined by GC/GC-MS using a standard curve with 1 µL chlorobenzene internal reference. [d]Heated to 50°C in the photoreactor.

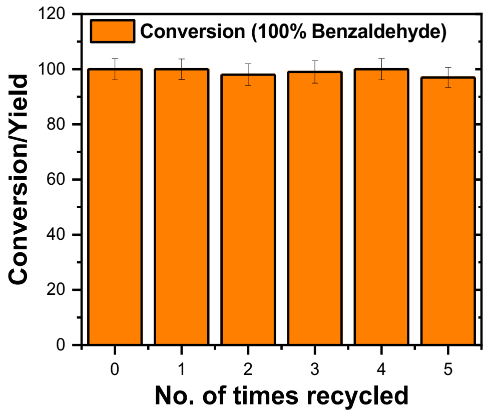

Figure S32. Recycle experiments.

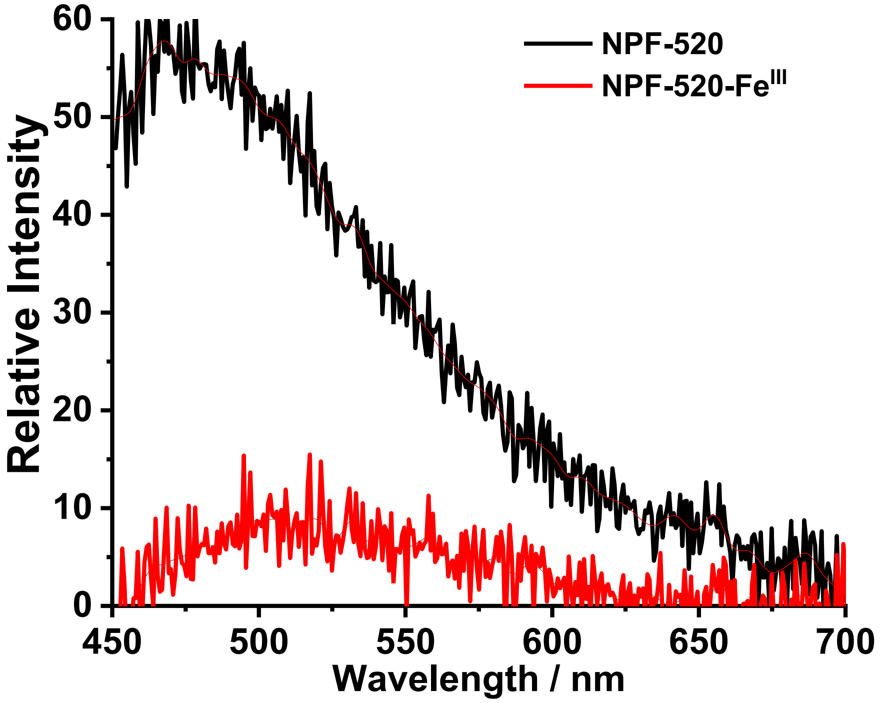

Figure S33. Fluorescence spectra of NPF-520 and NPF-520-Fe^{III} in solid state (excitation wavelength was 395 nm). The absolute quantum yields were 5.4% and 0.9%, respectively.

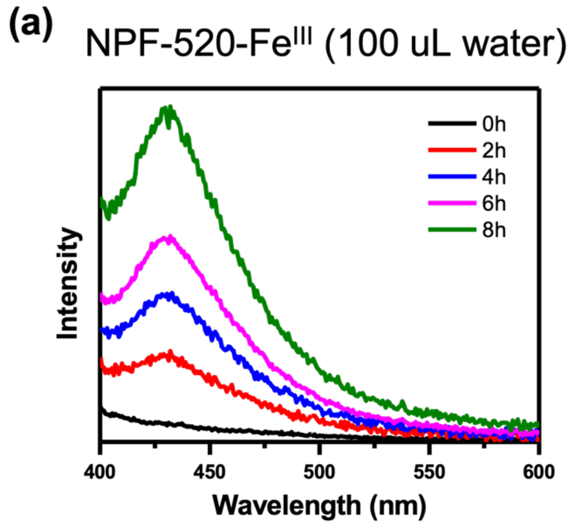

Figure S34. Fluorescence of oxidized BDC with time. Peak appears at 430 nm as BDC is oxidized by hydroxyl radical •OH.

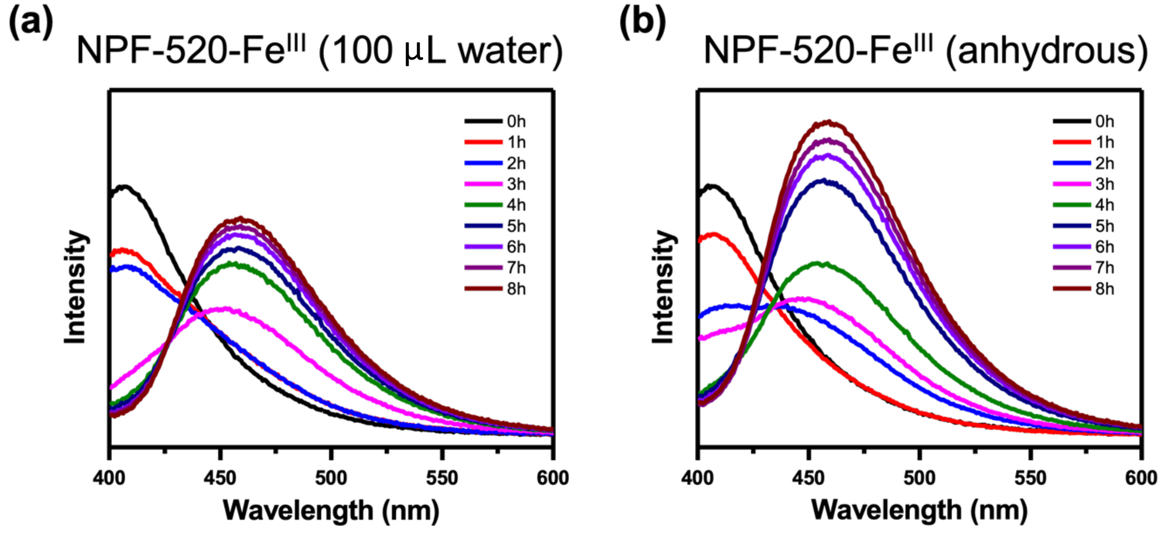

Figure S35. Fluorescence of luminol with time. Peak shifts from 410 nm to 460 nm as luminol is oxidized by superoxide $O_2^{\bullet -}$.

Table S6. Photocatalytic toluene oxidation in the presence of quenching agents.

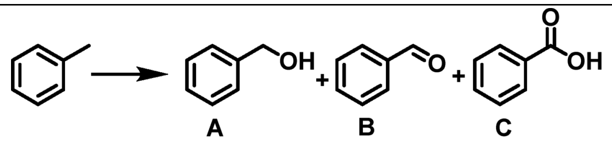

Entry	Catalyst	Additive	Conversion[c]	Selectivity[c] (A/B/C)
1[a]	NPF-520-Fe^{III}	2-methylfuran	100	0/0/100
2[a]		DABCO	100	0/0/100
3[a]		TEMPO	0	-
4[a]		triethylamine	0	-
5[a]		$(NH_4)_2Ce(NO_3)_6$	73	6/91/3
6[ad]		H_2O_2	4	0/100/0

[a]Reaction conditions: 5 μL toluene, 1 mL MeCN, 5 mol% catalyst (based on Fe^{III}), 395 nm blue LED photoreactor, 8 h, 1 atm O_2. [b]Reaction conditions: 10 μL toluene, 1 mL MeCN, 1 mol% catalyst (based on Fe^{III}), 395 nm blue LED photoreactor, 3 h, 1 atm O_2. [c]Determined by GC/GC-MS using a standard curve with 1 μL chlorobenzene internal reference. [d]1 atm N_2 instead of O_2.

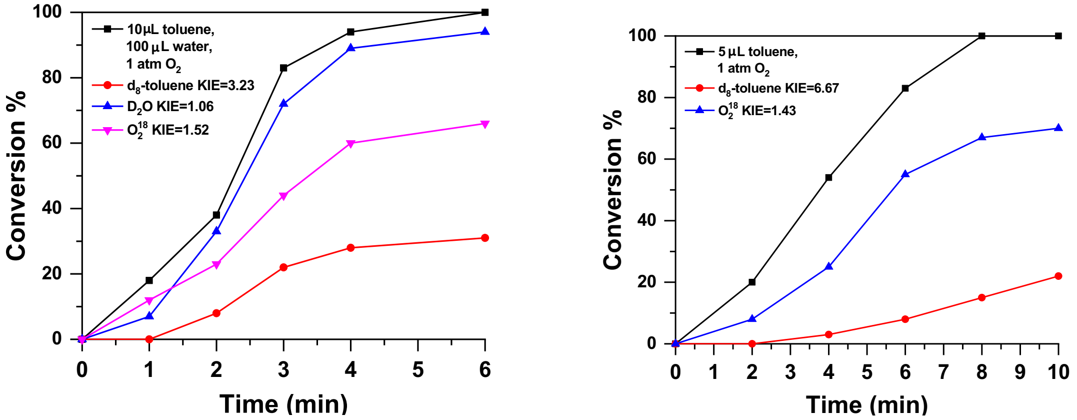

Figure S36. Kinetic plots and KIE of toluene oxidation by NPF-520-Fe^{III} in the presence of water and under anhydrous conditions.

Section S-11 References

(1) Zhang, M.; Chen, Y. P.; Bosch, M.; Gentle, T., 3rd; Wang, K.; Feng, D.; Wang, Z. U.; Zhou, H. C. Symmetry-guided synthesis of highly porous metal-organic frameworks with fluorite topology. *Angew. Chem. Int. Ed.* **2014**, *53* (3), 815-818. DOI: 10.1002/anie.201307340.

(2) Grosjean, S.; Hassan, Z.; Wöll, C.; Bräse, S. Diverse Multi-Functionalized Oligoarenes and Heteroarenes for Porous Crystalline Materials. *Eur. J. Org. Chem.* **2019**, *2019* (7), 1446-1460. DOI: 10.1002/ejoc.201801232.

(3) Ku, C.-H.; Kuo, C.-H.; Chen, C.-Y.; Leung, M.-K.; Hsieh, K.-H. PLED devices containing triphenylamine-derived polyurethanes as hole-transporting layers exhibit high current efficiencies. *J. Mater. Chem.* **2008**, *18* (12), 1296-1301. DOI: 10.1039/b715929c.

(4) Yuan, Y.; Huang, H.; Chen, L.; Chen, Y. *N,N'*-Bicarbazole: A Versatile Building Block toward the Construction of Conjugated Porous Polymers for CO_2 Capture and Dyes Adsorption. *Macromolecules* **2017**, *50* (13), 4993-5003. DOI: 10.1021/acs.macromol.7b00971.

(5) Gómez-Gualdrón, D. A.; Colón, Y. J.; Zhang, X.; Wang, T. C.; Chen, Y.-S.; Hupp, J. T.; Yildirim, T.; Farha, O. K.; Zhang, J.; Snurr, R. Q. Evaluating topologically diverse metal–organic frameworks for cryo-adsorbed hydrogen storage. *Energy Environ. Sci.* **2016**, *9* (10), 3279-3289. DOI: 10.1039/c6ee02104b.

(6) *APEX2*; Bruker AXS Inc.: Madison, Wisconsin, USA, 2010. (accessed.

(7) *SAINT*; Data Reduction Software. Bruker AXS Inc.: Madison, Wisconsin, USA, 2009. (accessed.

(8) *SADABS*; Program for empirical absorption correction: University of Göttingen, Göttingen, Germany, 2008. (accessed.

(9) Sheldrick, G. M. Phase annealing in SHELX-90: direct methods for larger structures. *Acta Crystallogr. A* **1990**, *A46* (Copyright (C) 2012 American Chemical Society (ACS). All Rights Reserved.), 467-473, 10.1107/S0108767390000277. DOI: 10.1107/s0108767390000277.

(10) Sheldrick, G. M. A short history of SHELX. *Acta Crystallogr. A* **2008**, *A64* (Copyright (C) 2012 American Chemical Society (ACS). All Rights Reserved.), 112-122, 10.1107/S0108767307043930. DOI: 10.1107/s0108767307043930.

(11) *SHELXL-97, Program for Structure Refinement*; University of Göttingen, Göttingen, Germany, 1997. (accessed.

(12) Spek, A. L. Single-crystal structure validation with the program PLATON. *Journal of Applied Crystallography* **2003**, *36* (Copyright (C) 2012 American Chemical Society (ACS). All Rights Reserved.), 7-13, 10.1107/S0021889802022112. DOI: 10.1107/s0021889802022112.
